# Trade-off between branching and polarity controls decision-making during cell migration

**DOI:** 10.1126/sciadv.ads2734

**Published:** 2026-01-01

**Authors:** Jiayi Liu, Javier Boix-Campos, Jonathan E. Ron, Johan M. Kux, Magdalena E. M. Oremek, Adriano G. Rossi, Nir S. Gov, Pablo J. Sáez

**Affiliations:** ^1^ Department of Chemical and Biological Physics, Weizmann Institute of Science, Rehovot, Israel.; ^2^ Department of Physics, Yale University, New Haven, CT, USA.; ^3^ Cell Communication and Migration Laboratory, Institute of Biochemistry and Molecular Cell Biology, Center for Experimental Medicine, University Medical Center Hamburg-Eppendorf, Hamburg, Germany.; ^4^ Department of Physics, Technion, Haifa, Israel.; ^5^ Centre for Inflammation Research, Queen’s Medical Research Institute, University of Edinburgh, Edinburgh, UK.

## Abstract

Motile cells often face microenvironmental constraints and obstacles that force them to extend multiple protrusions. However, the analysis of shape dynamics during directional decision-making has been restricted to single junctions. Here, we combined live-cell imaging and a coarse-grained model to study the migratory behavior of highly branched cells while simultaneously facing several junctions. The theoretical model predicts that the choice of a new direction is determined by the competition between the cellular protrusions in the form of seesaw oscillations. We found that macrophages and endothelial cells display different regimes moving on hexagonal networks, despite sharing a mesenchymal (i.e., adhesion-dependent) migratory strategy. The model describes the motility of both cell types and reveals a trade-off between branching and speed: Having many protrusions allows local microenvironmental exploration for directional cues, but long-range migration efficiency improves with fewer protrusions. Collectively, our data highlight the relevance and provide insights for the regulation of shape dynamics during cell navigation in complex geometries.

## Teaser

Highly branched cells require fine-tuning of polarity and shape dynamics to ensure balance between exploration and migration.

## INTRODUCTION

Cells migrate within tissues during physiological and pathological processes, such as embryo development, angiogenesis, tissue growth and repair, immune response, and cancer propagation (*1*–*5*). Because of the constraints of the microenvironment, motile cells often need to navigate tissues and organs while avoiding surrounding obstacles (*6*, *7*). This response has been studied in in vitro models using different geometries and types of confinement (*8*–*19*) but mostly limited to single junctions. Once migrating cells encounter junctions, they generate protrusions along alternate directions (*20*) and eventually select a new direction in a process named directional decision-making (*5*, *8*, *12*, *21*). This behavior is found in different cell types, endothelial (*22*), immune (*23*, *24*), and cancer (*1*) cells. However, the migration of highly branched cells in complex networks has not been systematically studied experimentally or theoretically, and the mechanisms that allow highly branched cells to maintain efficient migration in complex geometries are not yet fully understood.

We have recently shown how membrane and cytoskeleton dynamics control directional decision-making of cells facing single symmetric Y-shaped junctions (*8*), characterized by deterministic seesaw oscillations in the protrusive activity between the competing cellular protrusions. Here, we generalize our theoretical model in single junctions (*8*) to describe cells that simultaneously span multiple junctions and are highly branched. This model fills a knowledge gap that has not been covered with previous theoretical and experimental models (*14*, *15*, *21*, *25*, *26*). Cellular branching frequently occurs for cells migrating within complex tissues. For example, during angiogenesis (*2*, *22*) and immune response (*27*), both endothelial cells and leukocytes are required to migrate within and over blood vessels, which often bifurcate and force cellular branching (*28*). In some cases, there is a bias along some paths (e.g., dead end, chemokine, etc.), but often there is no bias at the junction (*8*), and the migration is unbiased. Unlike other leukocytes, macrophages exhibit a mesenchymal migratory strategy that is highly dependent on adhesion (*27*, *29*), and this is also observed in endothelial cells (*2*, *30*). Thus, we used these two cell types as models to study branching dynamics during migration on complex networks.

We combined live-cell imaging with micropatterning and advanced image analysis based on machine learning to monitor the spontaneous migratory behavior and shape dynamics of bone marrow–derived macrophages and human umbilical vein endothelial cells (HUVECs) on adhesive hexagonal networks. We found that both macrophages and HUVECs effectively resolve high levels of branching to continue their migration, although macrophages were more efficient in this process. In addition, we present a coarse-grained theoretical model to describe how highly branched cells form competing arms and how they are able to maintain their migratory capacity. The model predicts the complex shape dynamics and migration characteristics of branched cells on hexagonal networks, when these cells span multiple junctions and have numerous competing protrusions. This goes beyond the previous description of a cell moving across a single junction (*8*) and describes the motility of both cell types, despite the differences in their migratory behaviors. Our model revealed an important trade-off that affects the optimal number of cellular branching: A larger number increases local sensing and microenvironmental exploration for directional cues, yet it decreases the efficiency of long-range cellular migration.

## RESULTS

### The model

**Fig. 1.**
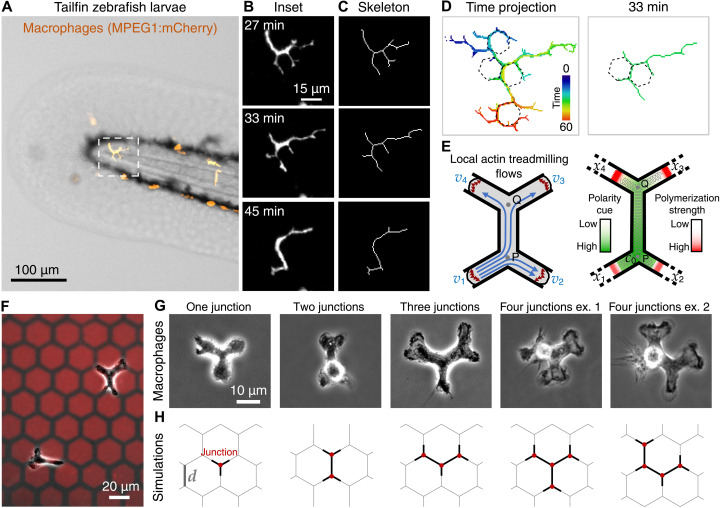
Experimental and theoretical models to reproduce the migration of highly branched cells observed in vivo. (**A**) Image of the tailfin of zebrafish larvae expressing a macrophage marker (MPEG1:mCherry, orange; movie S1). Dashed square indicates the region of the migrating macrophage shown in (B). (**B**) Fluorescent images of a macrophage migrating in vivo (movie S2). (**C**) Skeletonization of the macrophage in (B) shows the multiple arms (movie S2). (**D**) Temporal color code showing the trajectory of the skeletonized macrophage shown in (B) and (C). Note that the track reveals roughly hexagonal shapes that force the branching of the cell. Hexagons are depicted in the time projection and in a single time point as indicated. (**E**) Scheme showing the local actin treadmilling flows at each edge of the arms and the symmetrical split of the local actin flow that emanates from arm 1 at each junction, first in junction “P” and then in “Q”. The concentration field of the polarity cue that is affected by the advection flows (green scale) and the resulting polymerization strength at the tips of the arms (red scale) is shown. Cell elasticity is denoted by the gray springs. (**F**) Macrophages in a micropatterned hexagonal network. Scale bar, 20 μm. (**G**) Macrophages spanning single or multiple junctions in adhesive micropatterns as indicated. Two examples of macrophages spanning four junctions are shown (ex. 1 and 2). (**H**) Simulations showing cells spanning junctions as in (G).

To enunciate our model, we considered that some tissues cells have a patterned topology that induces the regular branching of motile cells. This example is the case of macrophages migrating in vivo in the basal layer of keratinocytes in the zebrafish tailfin (Fig. 1A and movie S1). Despite their morphological heterogeneity, these keratinocytes have a polygonal shape that often resembles hexagons (*31*), which forces macrophages to branch multiple times following the topology of the tissue (Fig. 1, B to D, and movie S2). Analysis of the cellular branching shows highly dynamic protrusions that are coordinated to allow proper macrophage tissue surveillance, while preserving migratory capacity (Fig. 1, B to D, and movie S2).

Our model is based on a cell composed of one-dimensional (1D) sections (*8*), which spontaneously self-polarize because of the coupling between the local actin polymerization activity at the leading edge of the protrusions (Fig. 1E). This coupling is mediated by the advection of an inhibitor of actin polymerization (termed “polarity cue”) by the net actin retrograde flow (Fig. 1E and fig. S1). The cell polarizes when, at steady state, the local polymerization activity is maintained as the largest in one of the cellular protrusions.

To mimic the shapes observed in vivo (Fig. 1D), we used adhesive micropatterns to produce hexagonal networks on which cells move and span up to six protrusive arms simultaneously (Fig. 1, F to G, and fig. S2). The migratory behavior of these highly bifurcated cells is analyzed and predicted by our model.

The polymerization of actin at the cellular tips is converted into protrusive forces, which compete with elastic and friction forces. This force balance drives the extension/retraction of the cellular protrusions and causes the cell to migrate. For simplicity, we assume that all the forces that act on each cellular protrusion are localized at their tips (frontal edges). The global elasticity of the cell is treated as an elastic spring (Fig. 1E).

As shown previously (*8*), for a cell with *N* ≥ 3 arms, i.e., spanning across *N* − 2 junctions (Fig. 1E), the dynamics of the arm *i* are described by three variables: (i) its length *x_i_*, (ii) the fraction of active slip-bond adhesion *n_i_* at the leading edge, and (iii) the local actin treadmilling flow velocity *v_i_* at the leading edge (Fig. 1E). The dynamic equations for these variables are given by

$${\dot{x}}_{i}=\frac{1}{\Gamma_{i}}\left[v_{i}-k\left(L-1\right)\right]$$ (1)

$${\dot{n}}_{i}=r\left(1-n_{i}\right)-n_{i}\text{exp}\left[\frac{-v_{i}+k\left(L-1\right)}{f_{s}n_{i}}\right]$$ (2)

$${\dot{v}}_{i}=-\delta \left(v_{i}-v_{i}^{*}\right)+\sigma \xi_{t}$$ (3)

In Eq. 1, Γ*_i_* is a nonconstant friction coefficient that depends on the direction of motion of the arm’s leading edge, given by

$$\Gamma_{i}=\Theta \left(\dot{x_{i}}\right)+\left[1-\Theta \left(\dot{x_{i}}\right)\right]n_{i}\;\kappa \;\text{exp}\left[\frac{v_{i}-k\left(L-1\right)}{f_{s}n_{i}}\right]$$ (4)

where Θ is a Heaviside function and κ is the effective spring constant of the bond linkers. When the arm is extending, the friction acts as a constant drag Γ*_i_* = 1, while when the arm is retracting, the friction is due to the adhesion of the slip bonds.

Equation 1 is a simplified description of the protrusive traction forces, which can be further elaborated to include the adhesion dependence of these forces (*32*). The restoring force of the cell elasticity in Eqs. 1 and 2 is described by a simple spring term (Fig. 1E), where *k* is the effective elasticity of the cell. In Eq. 2, *f*_s_ describes the susceptibility of the slip bonds to detach because of the applied force, and *r* is the effective cell-substrate adhesiveness.

In Eq. 3, δ is the rate at which the local actin flows relax to the steady-state solutions *v_i_*^∗^, which is given by (*33*)

$$v_{i}^{*}=\beta \frac{c_{s}}{c_{s}+c_{i}\left(x_{i}\right)}$$ (5)

where *c_i_*(*x_i_*) is the concentration of actin polymerization inhibitor at the tip of arm *i* (at the coordinate *x_i_*), *c*_s_ is the saturation concentration, and β is the maximal actin polymerization speed at the arm edges (hereafter referred to as “actin activity”). The term σξ_t_ in Eq. 3 describes the noise in the actin polymerization activity, modeled as a Gaussian noise with amplitude σ.

The calculation of the spatial distribution of the inhibitor concentration along the different arms *c_i_*(*x_i_*) was performed (fig. S1B). The spatial distribution is composed of exponential sections, maintaining continuity at the junctions, a no-flux boundary condition, and a constant total amount of inhibitor within the cell. The total length of the cell is given by

$$L=\sum_{i}x_{i}+\left(N-3\right)d$$ (6)

where *d* is the distance between two adjacent junctions on the network (Fig. 1H). Following the initialization of *x_i_*, *n_i_*, and *v_i_* of each arm, their temporal evolution during the migration process can be obtained through numerical integration of Eqs. 1 to 3. In this study, we used the symmetric initial condition.

Let us make a note regarding the choice of model parameters. For simplicity, we kept many of the model parameters at the values that we previously calibrated (*8*) to the behavior of HUVECs, given in table S1 (see section S1). The remaining free parameters that we vary are as follows: β, the actin activity; σ, the local noise in the actin activity at the tips of the cellular protrusions; *k*, the elastic stiffness of the cell (including effects of contractility); and *r*, the cell-substrate adhesiveness. For the HUVECs, we use for these model parameters the values that were calibrated previously (*8*). There is a large variability between cells, even within the same cell type, so the values we use here are merely representative. For the macrophages, we fit parameters that correspond to higher cytoskeletal activity, larger actin activity β, and actin noise σ. This is in accordance with our previous observation that highly motile cells, such as cancer cells, are distinguished by having higher values of β (*8*).

### Increased branching decreases cell polarity and migration

**Fig. 2.**
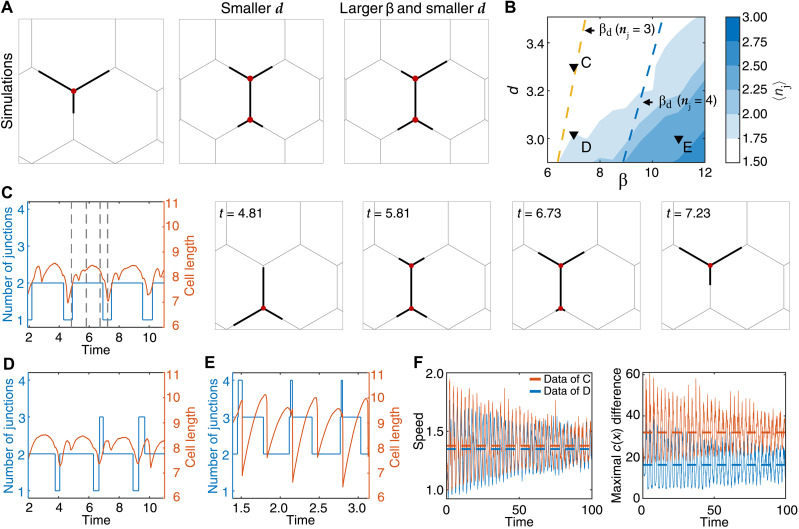
Cellular polarity depends on the number of arms (junctions) in the model. (**A**) Simulation examples of cells with the chosen parameters of the model (β = 7.0, *d* = 3.3), or with a smaller *d* (β = 7.0, *d* = 3.0), or a larger β and smaller *d* (β = 11.0, *d* = 3.0). (**B**) The β*-d* phase diagram of the mean number of junctions spanned by the cell (*n*_j_) (color bar). Yellow and blue dashed lines denote the critical (minimal) β_d_ for cells to be able to span three and four junctions, respectively (see fig. S1E and eq. S28). Selected regions are depicted with inverted triangles [(C) to (E)]. (**C**) Dynamics of the number of junctions and total cell length for sections (β = 7.0, *d* = 3.3; movie S3). Selected snapshots for the time stamps (gray dashed lines). (**D** and **E**) Graphs showing similar dynamics for different values of β and *d* [(D) β = 7.0, *d* = 3.0 (movie S3) and (E) β = 11.0, *d* = 3.0], respectively. (**F**) Center of mass (C.O.M.) speed (left) and the maximal difference between the polarity cue concentration at the arm tips [*c*(*x_i_*)] across the cell (right), comparing (C) and (D).

We run simulations to analyze the shape dynamics of a cell moving in a hexagonal network, as function of the actin activity parameter β and the grid size *d* (Fig. 2). We found that migrating cells have more arms when β is larger because the protrusive forces that elongate the cell are stronger and longer cells span more junctions (Fig. 2, A and B). Similarly, cells span more junctions and have more arms when *d* is smaller (Fig. 2, A and B, and movie S3).

We selected three regions from the phase diagram (black inverted triangles in Fig. 2B) to display the typical dynamics of the number of arms and the total cell length (Fig. 2B). By increasing β, we observed that the number of junctions spanned by the cell is increased because of the larger mean values and the larger fluctuations in the total cell length (Fig. 2B).

The critical β_d_, above which the cell extends to a length that spans *n*_j_ junctions, naturally increases with *n*_j_ (Fig. 2B and fig. S1F). We found that this critical β_d_ can be larger than the minimal value needed to allow the cell to polarize and leave the junction, β_c_ (see section S2). When β_c_ > β_d_, the cell becomes trapped across two junctions (shaded region in fig. S1F). Similarly, the length of cell trapped while spanning *n*_j_ junctions depends on the model parameters (section S2). This is due to the increased competition between the leading edges, which makes it more difficult for the cell to polarize as the number of arms (junctions) increases. Next, we analyzed the relation between the number of arms and migration speed (Fig. 2, C to E). We found that at the same value of β and thus with very similar instantaneous cell speed and length (Fig. 2F and movie S3), the cell polarity is substantially larger (Fig. 2F) when the number of arms is smaller, for example, on the hexagonal network of larger dimensions. These results expose a trade-off for the migration of branched cells on complex networks: A larger number of junctions allow cells to better explore their local microenvironment and become more sensitive to weak directional cues. On the other hand, an increased number of junctions decrease cellular polarization and more likely to decrease their persistence as well, limiting their ability to explore larger spatial domains.

**Fig. 3.**
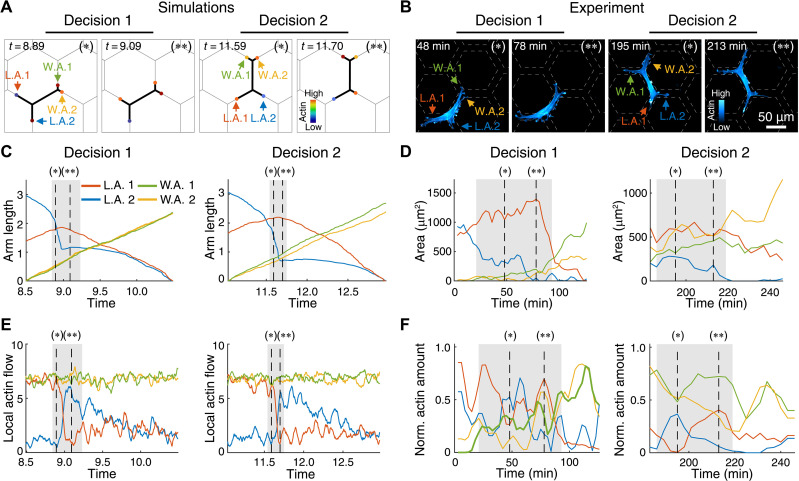
Deterministic seesaw oscillations in the competing arms define directional decision-making. (**A**) Two decision-making events are shown in a simulation of a migrating HUVECs (movie S4). Before and after the decision are shown with * and **, respectively. Key parameters: β = 7.0, *d* = 3.2, σ = 1.0, *k* = 0.8, and *r* = 5.0. Cellular arms are labeled to depict the winning and losing arms, W.A. and L.A., respectively. (**B**) Similar to the simulation in (A), microscopy images of a migrating HUVECs expressing F-tractin (cyan) showing F-actin dynamics (movie S5). Arms and decision events are labeled as in (A). (**C**) Graphs showing arm length dynamics in a simulation. (**D**) Similar to (C), graph showing tip area dynamics of the migrating HUVECs shown in (B). Two decisions are also shown. Note the similar results obtained with the model and the experiment. (**E** and **F**) Dynamics of local actin flow in the simulation (E) and the normalized actin activity in the live-cell experiment (F).

To compare our model predictions with experiments, we performed live-cell imaging of HUVECs and bone marrow–derived macrophages moving on hexagonal networks of adhesive stripes, as the migration of both cell types is dependent on adhesion (*27*, *29*). The size of the hexagonal grid was adapted to each cell to ensure high branching (fig. S2). Guided by the simulation results (Fig. 3A and movie S4), we monitored branch and actin dynamics at the tips of the cellular protrusions during HUVEC migration (Fig. 3B and movie S5). We treated the area of the actin-filled tips of the arms as a measure of the actin protrusive activity, as previously shown (*8*). The experimental observations confirm the model predictions, as shown by the example of two decisions (Fig. 3, C to F). Moreover, we identified seesaw oscillations in the actin protrusive activity at the tips of the losing arms during the migration and directional decision-making of the branched cell (Fig. 3, C to F). The agreement between experiments and simulations verifies a central prediction of the model, the deterministic seesaw oscillations in the protrusive activities at the tips of competing arms, thus lending further support for the basic underlying mechanism of the model. We similarly observed the seesaw oscillations in the simulations for the parameters corresponding to macrophage migration (fig. S3).

**Fig. 4.**
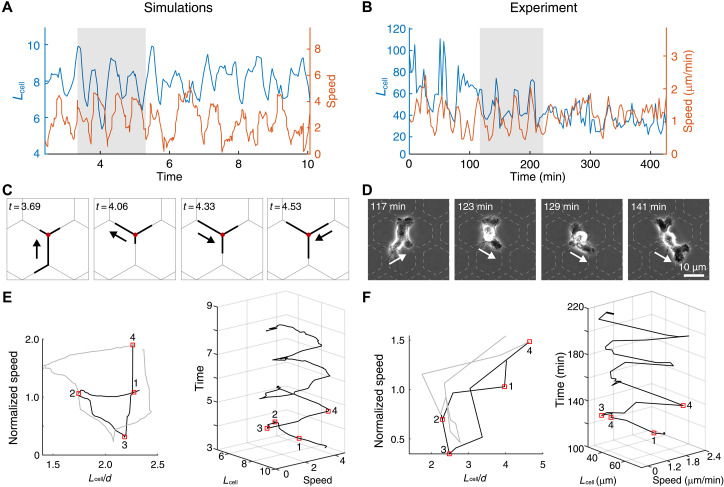
Length-speed cycles during migration of branched cells. (**A**) Dynamics of the cell length and C.O.M. speed in a simulation of a migrating macrophage (movie S6). (**B**) Similar dynamics in an experiment of a migrating macrophage (movie S7). (**C** and **D**) Selected snapshots of a simulation and cell at selected time points during the oscillation. Key parameters: β = 10.0, *d* = 3.8, σ = 3.0, *k* = 0.8, and *r* = 5.0. (**E** and **F**) Typical oscillation (black line) and the following oscillation (gray line) in the *L*_cell_-*v*_C.O.M._ space. 3D trajectories plotted with cell length as the *x* axis, C.O.M. speed as the *y* axis, and time as the *z* axis. Red boxes mark time points corresponding to those highlighted in (C) and (D).

Then, we focused on the oscillatory patterns of cell length and speed [of the cellular center of mass (C.O.M.)] during migration on the network (Fig. 4). Despite the noisy nature of these oscillations, our model revealed a typical cycle-like pattern (Fig. 4A and movie S6), which is also found in the experiments (Fig. 4, B and F). The cycle starts with an elongated cell (spanning more than one junction) that is highly polarized and migrates smoothly at some finite speed (1), until it snaps (stick slip) to a shorter length after losing grip of a junction (2). Then, the cell loses its polarity and elongates rather uniformly, which leads to lower value of its directional speed (3). Next, the cell elongates over the next junction increasing its polarity and speed (4), completing the cycle. As abovementioned, we found very similar cycles in the experimental observations of macrophages moving on the network (Fig. 4, B, D, and F, and movie S7).

The theoretical model makes detailed predictions regarding cell shape dynamics, such as length and number of arms, and relates these features to the cell speed and the underlying actin activity at the cellular tips, including the emergence of deterministic seesaw oscillations. The model predicts that increasing number of arms decreases the polarity of the branched cell. In the next section, we describe the consequences of this branching-polarity trade-off on the large-scale migration of branched cells on networks.

### Large-scale migration characteristics of branched cells

**Fig. 5.**
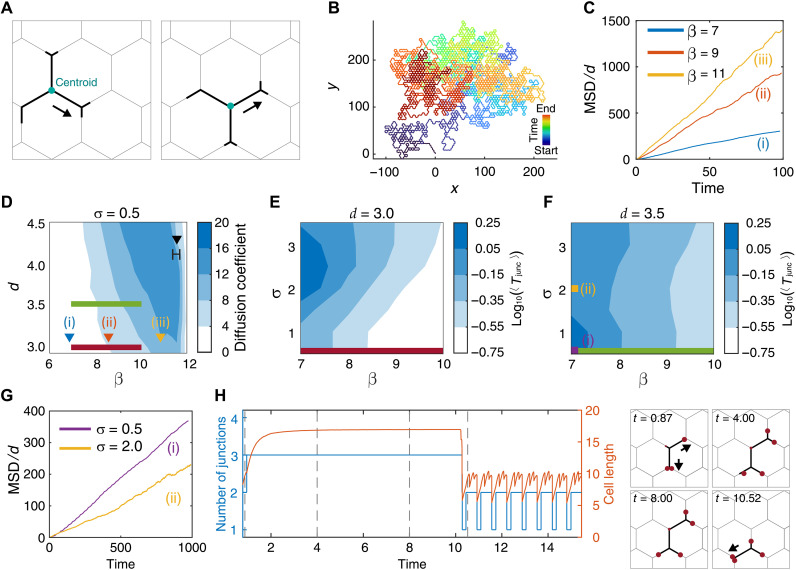
Large-scale migration patterns of cells on networks predicted by the model. (**A**) Example of centroid detection in a simulation. (**B**) Representative C.O.M. of a long trajectory for β = 9, *d* = 3.1, and σ = 0.5. Maximal simulation time: *T* = 10,000. (**C**) MSD time series for β = 7, 9, 11, *d* = 3.1, and σ = 0.5. (**D**) β-*d* phase diagram of the diffusion coefficient of the cellular C.O.M. (*D*_c_; Eq. 7) for σ = 0.5. Colored lines correspond to ranges marked in (E) and (F); colored inverted triangles match cases in (C). Maximal simulation time: *T* = 100. (**E** and **F**) β-σ phase diagram of (*T*_junc_, log scale) for *d* = 3.0 and *d* = 3.5, respectively. (**G**) MSD time series for two noise levels of the phase diagram [purple/yellow squares in (F)] for β = 7 and *d* = 3.5. (**H**) Dynamics of number of junctions and cell length during a slow-mode event for β = 12.5, *d* = 4.3, and σ = 0.5, marked by the black inverted triangle in (D). Snapshots at time points marked by dashed lines in the graph. Red circle size indicates actin flow at arm tips. Arrows indicate branch growing. Other key parameters: *k* = 0.8 and *r* = 5.0.

Next, we investigated the large-scale migration features of the cells moving on the hexagonal networks. These features include the residency time distribution at the junctions and the overall migration distance. We monitored the C.O.M. in the simulations (Fig. 5, A and B) and calculated the mean square displacement (MSD) (Fig. 5C and section S3). We plot the time series of MSD for different values of β and fit them using the power-law function MSD = *Kt*^α^ (Fig. 5C). We found that α ≈ 1 for all the sets, corresponding to regular diffusive motion, which is depicted by a typical trajectory of cellular C.O.M. during a long run (Fig. 5B, β = 9, *d* = 3.1).

For this 2D Brownian motion, the MSD is

$$\text{MSD}\left(t\right)=4D_{c}t$$ (7)

where *D*_c_ is the diffusion coefficient of the cell trajectory along the hexagonal lattice, calculated using the linear fit to the time series of the MSD. We found that *D*_c_ increases proportionally with β and *d* (Fig. 5D) for β < 11.5. This is expected because in single junctions, the residency time (which is defined as the time it takes a cell to leave a junction since it was first occupied) of cells at the junction generally decreases with increasing actin activity β (*8*). Similarly, we study the junction residency time of the cell (*T*_junc_), as function of β and σ, which is the cellular internal noise (Eq. 3 and Fig. 5, E and F). We used different grid sizes (*d* = 3.0 and *d* = 3.5) and found that the average residency time ⟨*T*_junc_⟩ decreases as β increases (Fig. 5, E and F). This inverse relation is consistent with single junction case (*8*), in which the actin activity at the edges of the arms increases the migration speed and decreases the residency time on a junction. This explains the role of β in increasing the cellular diffusion (Fig. 5D).

By increasing grid size *d*, the diffusion coefficient increases (Fig. 5D) because of the decrease in the number of junctions that the cell spans (Fig. 2B). By decreasing the number of junctions that the cell spans, we increase the polarity of the cell (Fig. 2F), as there are fewer competing leading edges. Polarity robustness gives rise to persistent migration, leading to higher diffusion coefficient.

For the network with *d* = 3.0, we found that the mean residency time ⟨*T*_junc_⟩ increased, as the noise amplitude σ increases (Fig. 5E). The residency time increases with the noise, as the strength of the flows that polarize the cell decreases. We analyzed the mean residency time of the cell over a specific number of junctions, $\langle T_{\text{junc}}^{n_{j}}\rangle$, where *n*_j_ represents the number of junctions that the cell spans (fig. S4). Cells that span two junctions display two peaks with low and high residency time $T_{\text{junc}}^{n_{j}=2}$ (fig. S4, A and B) as a consequence of short time intervals when *n*_j_ transits from two to three and the long time interval when *n*_j_ transits from two to one (Fig. 2D). At high noise, the residency time distribution is exponential with a long tail (fig. S4C). These results in which noise increases the residency time were also found for cells moving in a single junction (*8*).

For a hexagonal grid of larger dimensions, *d* = 3.5, we find that moderate noise can decrease the average junction residency time (Fig. 5F). This trend is driven by the dynamics of the two-junction spanning cells (fig. S4B). Because of the larger grid size, the cells form shorter arms that extend at the ends of the spanned junctions, and therefore noise can drive these short arms to lose their “grip” on one of the junctions (fig. S4, G and H). The corresponding simulation snapshots are shown (fig. S4, G and H). This behavior is unique to cells spanning multiple junctions, whereby an increase in cellular noise decreases junction occupation time and increases the frequency of hopping between junctions. However, the reduction in junction residency time does not correspond to faster cellular diffusion motion because the MSD for high noise is substantially smaller than for low noise (Fig. 5G). The shorter junction residency time is driven by short bouts of the cell spanning two junctions and then losing its grip on one of these junctions, a form of rapid oscillations around the same average location, and therefore does not correspond to faster overall migration.

At larger values of β, a rapid decrease in the diffusion coefficient of the cellular motion was observed (Fig. 5D, see point denoted H). This is attributed to the occurrence of a “slow process,” in which the cell tends to form protrusions of approximately equal length, substantially increasing the time the cell spends at the junctions. This phenomenon was also observed in the single junction case (*8*). In addition, we found that the diffusion coefficient *D*_c_ decreased at large β and large *d*, when the cell is in the slow-mode regime (fig. S4E). In an example of the slow process (β = 12.5, *d* = 4.3), we plot the number of junctions and cell length as functions of time (Fig. 5H). In addition, in the snapshots, we show the local actin flow velocity at the arm tips represented by the size of the red circles. In addition, we plotted the distributions of $T_{\text{junc}}^{n_{j}}$ for this set of parameters (fig. S4F). These results reveal that the slow process, first identified in the limit of large actin activity (β) for a single junction (*8*), also occurs for highly branched cells, even when the cell has secondary branches (Fig. 5H). We therefore conclude that while more active (larger β) cells migrate faster and diffuse more efficiently on the network, above some threshold activity they get increasingly trapped by “slow-mode” events. Comparing the dynamics of macrophages to the model, we predicted that this cell type has β ≈ 10, which is lower than the slow-mode regime (Fig. 5D). Nevertheless, individual macrophage cells may have activity that is large enough to occasionally exhibit a weak form of the slow-model event (fig. S5). An alternative way to quantify the migration over the network is the “hexagonal residency time” of the cells, *T*_hex_, which is defined as the time from when the C.O.M. of the cell enters a hexagonal region, until it leaves it entering a new one (section S6 and fig. S6).

To validate our model with experiments, we compared the calculated dynamics of the cell shape (number of junctions, cell length, and arm lengths) and the distributions of junction residency time measured in our experimental observations. To measure these parameters in our experimental data, we used a machine learning–based image analysis to compute the positions of the tips of cellular arms that emanate from the junctions spanned by the cells. To facilitate clear comparison between the experiments and the theoretical model, we normalized the measurements from both experiments and simulations by the grid size *d* and by the mean residency time of the cell when it covers the smallest number of junctions (depending on the condition), respectively.

**Fig. 6.**
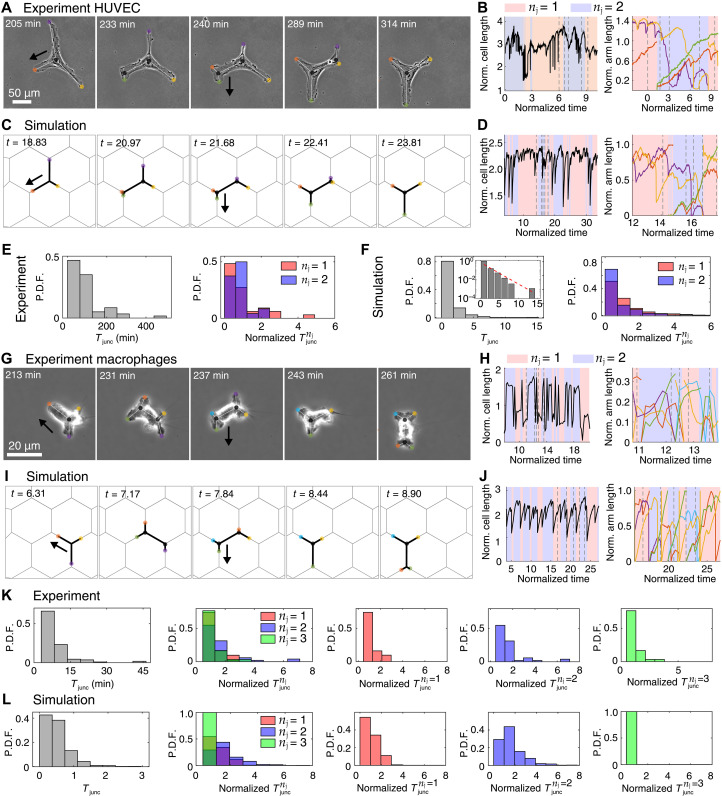
Different adhesion-dependent migration regimes in HUVECs and macrophages. (**A**) Experiment on a migrating HUVECs (movie S8). (**B**) Dynamics of the normalized cell and arms lengths. Dashed lines correspond to the selected microscopic images in (A). (**C**) Simulation of a migrating HUVECs (movie S9). Key parameters: β = 7.0, *d* = 3.6, σ = 2.0, *k* = 0.8, and *r* = 5. (**D**) Graphs similar to those shown in (B) but for the simulation in (C). (**E**) P.D.F. of *T*_junc_ and normalized $T_{\text{junc}}^{n_{j}}$ in the HUVEC experiments. (**F**) Distribution (P.D.F.) of *T*_junc_ and distribution (P.D.F.) of $T_{\text{junc}}^{n_{j}}$ from a long simulation with the parameters of (D). The inset shows a semilog plot depicting that the distribution is exponential. (**G**) Experiment of a migrating macrophage (movie S7). (**H**) Dynamics of the normalized cell and arm lengths. Key simulation parameters: β = 10.0, *d* = 3.8, σ = 3.0, *k* = 0.8, and *r* = 5.0. (**I**) Simulation of a migrating macrophage (movie S10). (**J**) Graphs similar to (H) but for the experiment in (I). (**K**) Distribution (P.D.F.) of *T*_junc_ and distribution of $T_{\text{junc}}^{n_{j}}$ in the experiments of macrophages, where the cells spans one, two, or three junctions. (**L**) Distribution (P.D.F.) of *T*_junc_ and distribution (P.D.F.) of $T_{\text{junc}}^{n_{j}}$ from a long simulation with parameters of (J).

Migrating HUVECs oscillated between spanning one and two junctions (Fig. 6, A and B, and movie S8). Comparison with the simulations shows that the cells exhibit very similar dynamics (Fig. 6, C and D, and movie S9). Some HUVECs also exhibited frequent fluctuations between one and two junctions or stable dynamics spanning two junctions (fig. S7, A to D, and movies S9 and S13 to S16). To capture the observed variations in cell shape and migration speeds between individual HUVECs, we slightly changed one of the cellular parameters in the model (since the variation in the micropattern dimensions are negligible, ∼1 to 5%). We chose to vary the cell elasticity parameter *k*, since it affects the total length of the cell and consequently the cell shape and the number of junctions it spans on the hexagonal network (Fig. 6D; fig. S7, B and D; and movies S9, S14, and S16). A similar change in dynamics arises if the hexagonal network has a slightly different size *d* (see simulation results in section S5 and fig. S4). The probability distribution function (P.D.F.) of the normalized junction residency time in both HUVEC experiments and simulations has a broad, exponential-like form (Fig. 6, E and F). The comparison of the distributions from the simulations at different noise levels (fig. S4, B and C and G and H) suggests that these cells are in a regime of large internal noise. Together, these data (Fig. 6, A to F, and movies S8 and S9) show good qualitative agreement between the model and the experiments in HUVECs.

Macrophages efficiently explored the hexagonal lattice, as the number of junctions spanned oscillated between one and three (Fig. 6, G and H, and movie S7). In comparison to HUVECs, macrophages exhibited shorter junction residency times, with a narrow distribution. This suggests that these cells are in a regime of higher overall actin protrusive activity (the parameter β in the model) and lower relative internal noise (σ relative to β; Fig. 6, I and J, and movie S10). These properties lead to a higher polarization of macrophages in comparison to HUVECs, which is expected for immune cells that need to efficiently migrate in complex microenvironments (*22*, *34*). We found in both experiments and simulations the same patterns of the junction residency time, with relatively short residency times for both *n*_j_ = 1 and 3 and the widest distribution extending to long residency times for *n*_j_ = 2 (Fig. 6, K and L). These patterns arise in our model as it follows: Short cells that span a single junction are highly polarized elongate quickly and migrate, leading to short $T_{\text{junc}}^{n_{j}=1}$. When cells are highly stretched and span three junctions, elastic restoring forces do not allow them to maintain this elongation for long durations, leading also to short $T_{\text{junc}}^{n_{j}=3}$. Last, when the cells are spanning two junctions, their polarization is weaker compared to *n*_j_ = 1 due to more numerous protrusions, and combined with the noise, it gives rise to a broad distribution of $T_{\text{junc}}^{n_{j}=2}$ (fig. S4C).

We next compared the results of simulations and experiments of HUVECs that are stuck at the junctions (fig. S8 and movies S19 to S22) and are not globally motile over the timescale of the experiment. In the simulations, this behavior occurred when the internal noise was too large for cells to efficiently polarize and migrate away from the junction. Overall, these observations are consistent with our model: Cells, such as HUVECs, that exhibit shape dynamics that correspond to lower actin activity (β parameter in our model) and higher relative internal noise, also exhibit lower migration and higher tendency to get trapped on junctions, especially when spanning more than one junction. Cells that correspond to higher actin activity, such as macrophages, exhibit efficient migration even when having highly bifurcated shapes.

**Fig. 7.**
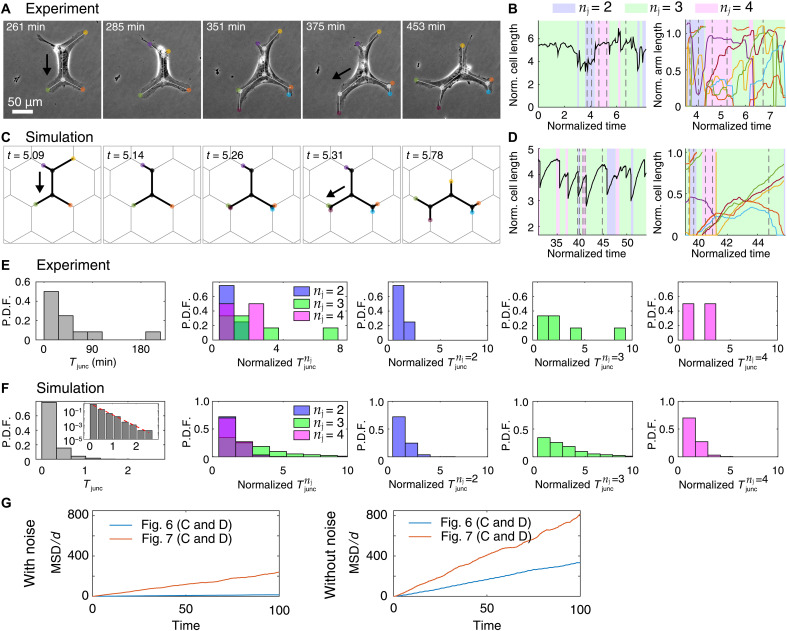
Large cell-substrate adhesiveness increases local exploration but reduces migration. (**A**) Microscopic images of a migrating HUVECs treated with 50 μM PD150606, a calpain inhibitor (movie S11). (**B**) Dynamics of the total cell length and the arm lengths of the experiment in (A). Gray dashed lines denote the snapshots of (A). (**C**) Simulation of HUVECs with larger cell-substrate adhesiveness (movie S12). Key parameters: β = 10.0, *d* = 2.4, σ = 2.7, and *r* = 7.0. (**D**) Graphs similar to those shown in (B) but for the simulation of (C). (**E**) Distribution (P.D.F.) of *T*_junc_ and distribution of $T_{\text{junc}}^{n_{j}}$ in the experimental regime in which the cells spans two, three, or four junctions. One cell across 576 min was used to obtain these distributions. (**F**) Distribution (P.D.F.) of *T*_junc_ and distribution of $T_{\text{junc}}^{n_{j}}$ of a long simulation with parameters of (D). Inset shows a semilog plot depicting that the distribution is exponential. (**G**) Comparisons of MSD of the simulated HUVECs of Fig. 6 and this figure, with the noise levels identical to those used in the corresponding simulations or without the noise.

We next tested the effect of substrate adhesion in our model. Mesenchymal migrating cells require firm adhesion to the substrate by forming focal adhesions, which are then disassembled at the cell rear by calpain (a Ca^2+^-dependent protease) (*2*, *22*, *30*). To increase cell adhesion, we prevented focal adhesion disassembly using an inhibitor of calpain (50 μM PD150606), which resulted in increased cell length of HUVECs (Fig. 7, A and B, and movie S11). Moreover, inhibition of focal adhesion disassembly slowed the cell rear detachment, and the elongated cells spanned a larger number of junctions, oscillating between two and four (Fig. 7B and movie S11). Similar results were obtained in the simulations by increasing the adhesion strength (the parameter *r* in our model; Eq. 2) and both the overall actin activity (β) and its noise (σ) compared to the normal HUVECs (Fig. 6, A to D, and Fig. 7, C and D). These changes have the effect of increasing the cell length and number of spanned junctions (Fig. 7, C and D; fig. S9; and movies S12 and S25), similar to the shape characteristics observed in the experiments. In addition, we tested an alternative inhibition of calpain by activating adenosine receptors, which leads to protein kinase A (PKA) activation (*35*), and consequently increased adhesion in both HUVECs and macrophages (fig. S10). Consistent with our findings in HUVECs, we observed that calpain inhibition with adenosine analog NECA (5′-*N*-ethylcarboxamidoadenosine) or the calpain inhibitor resulted in increased cell length and reduced migratory efficiency of macrophages moving in the hexagonal network (fig. S11), as shown for HUVECs.

The agreement between the experiments and simulations also extends to the patterns of the junction residency time, with relatively short residency times for both *n*_j_ = 2 and 4 and the widest distribution extending to long residency times for *n*_j_ = 3 (Fig. 7, E and F). These patterns in the residency times can be explained in our model: Short cells spanning two junctions have few arms and are therefore strongly polarized and migrate quickly, leading to short $T_{\text{junc}}^{n_{j}=2}$. When cells are highly stretched and span four junctions, elastic restoring forces do not allow them to maintain this elongation for long durations, leading also to short $T_{\text{junc}}^{n_{j}=4}$. Last, when the cells are spanning three junctions, their polarization is weaker compared to *n*_j_ = 2 due to more numerous protrusions, and combined with the noise, it gives rise to a broad distribution of $T_{\text{junc}}^{n_{j}=3}$ (fig. S4C).

In addition, we show the calculated effects of the changes induced by the drug on the large-scale diffusion of the simulated cells (Fig. 7G). We compared the normalized MSD of the HUVECs (Figs. 6D and 7D), with and without the actin polymerization noise (see also fig. S9C). As expected, the diffusion decreases for lower β and higher adhesion, even without noise, due to the cell speed decreasing. However, large noise has an additional dominant effect that can lead to long residency times due to the cells getting trapped over multiple junctions and therefore resulting in a marked decrease in the diffusion coefficient *D*_c_ (Eq. 7) over the network. These comparisons between the model and experiments demonstrate that the model correctly predicts the global characteristics of the branched cell migration on the networks (Figs. 6 and 7). These results expose a trade-off between the number of arms (which can help cells better explore their surroundings) and the ability of cells to maintain persistent migration. This trade-off has important consequences for the functionality of immune cells, for example.

**Fig. 8.**
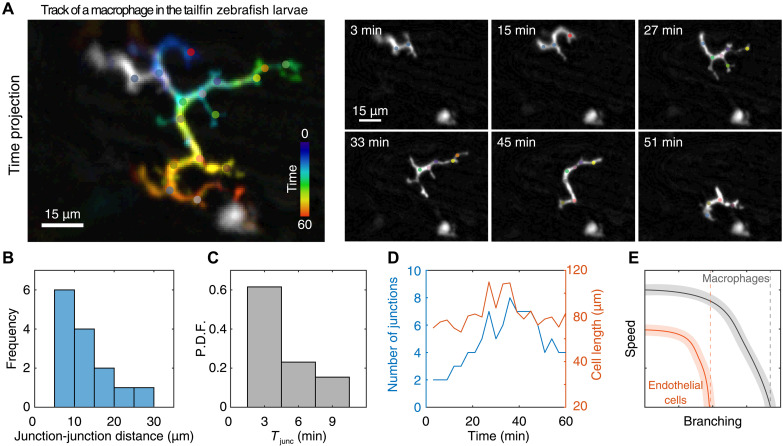
Macrophage migration in vivo. (**A**) Temporal color code (time projection) showing the trajectory of a macrophage migrating in the zebrafish tailfin (movie S2). Junctions are depicted with colored dots. This label is the same for the single panels. Snapshots of a migrating macrophage at indicated times. (**B**) Distribution of the distance between each pair of adjacent junctions. (**C**) Distribution (P.D.F.) of the residency time (*T*_junc_) of macrophages migrating in vivo. (**D**) Dynamics of the number of junctions and total cell length across time. (**E**) Schematic illustration of the trade-off between the number of branching and cell speed (polarity) for endothelial cells and macrophages. Dashed line denotes the value at which speed reach zero value. Note that immune cells are able to maintain their migration even when highly branched.

Last, to provide an in vivo validation of our model, we analyzed the spontaneous migration of interstitial macrophages moving in the zebrafish tailfin. As described above, we found that macrophages have branched shapes (Figs. 1 and 8A and movie S2), as they are embedded and move along the regular array formed by basal keratinocytes that have a polygonal shape (*31*), which is frequently hexagonal as aforementioned (Fig. 1A). Because of the cellular heterogeneity expected in a tissue, the shapes have different lengths of the sections between junctions (*d*; Fig. 8B), unlike the regular hexagonal networks of the in vitro experiments and simulations. However, the distances between the junctions are within a similar range of values compared to that used in the in vitro experiments. Similar to the observations made in the hexagonal micropatterns (Figs. 6 and 7), the junction residency time shows a tight peak (Fig. 8C). As expected, the number of junctions increases with the cell length (Fig. 8D), as obtained in both in vitro experiments and simulations (Figs. 6 and 7), showing that our model can be used to describe the shape dynamics and migratory behavior of cells moving in in vivo, such as macrophages.

## DISCUSSION

In this work, we present a theoretical framework and experimental proof of concept for the shape dynamics and migration of branched cells inside complex networks. We describe how migrating cells span multiple junctions and have a highly ramified shape with multiple competing arms in geometries such as those observed during cellular migration within tissues in vivo (*22*, *34*). The model describes the self-polarization of the cell due to the feedback between the actin activity at the leading edges of the cellular protrusions and the global redistribution of a polarity cue that acts to inhibit local actin polymerization and protrusive activity. This model extends our recent description of migration over a single Y-shaped junction (*8*), and it reveals an unknown trade-off between microenvironmental exploration for directional cues and long-range migration efficiency during directional decision-making.

Our model fills a gap in the theoretical understanding of cellular shape and migration dynamics. Previous models describe cells growing branches independently of each other but do not incorporate the intrinsic polarity competition between protrusions, and the reduction in speed for increasing number of protrusions is a consequence of force cancelation (*36*). On the contrary, our model incorporates an inhibitory polarity cue and allows us to predict the spontaneous out-of-phase oscillations in the activity and length dynamics of the protrusions, which are observed in the experiments.

We compare the cellular dynamics predicted by the theoretical model with the migratory behavior of two cell types that undergo stick-slip migration: HUVECs and macrophages, which use a mesenchymal, adhesion-dependent migratory strategy (*2*, *27*, *29*, *30*). The network dimensions were adapted to each cell: By constraining the cells to simultaneously span several junctions, we induced a high level of branching. The model provides a good qualitative description of the observed cellular shape dynamics.

Using our model, we investigated the role of noise in actin activity, and the results suggest that cells with overall weak and very noisy internal actin retrograde flows are susceptible to losing their polarity and getting trapped at the network junctions. This indicates that there is an optimal range for the number of protrusions during cell migration: On the one hand, these protrusions contribute to pathfinding by allowing the cells to better sense their local microenvironment for directional cues. On the other hand, if the number of protrusions is too high, then this will hinder efficient migration. This trade-off between microenvironmental sensing and efficient motility may determine the optimal levels of actomyosin contractility and protrusive activity for each cell type (Fig. 8E). Our data suggest that this trade-off is evident during the faster migration of macrophages in the hexagonal array, in comparison to HUVECs. This could be directly related to the surveillance function of macrophages, which requires the efficient exploration of the microenvironment, while preserving their migratory capacity (*34*).

Actomyosin contractility requires Ca^2+^ signals in the cytoplasm. Ca^2+^ signaling proteins are directly involved in migration and branching (*6*), suggesting a pivotal role during directional decision-making. Consequently, the manipulation of calcium-dependent protease calpain led to an increase in the cell length and a reduction in the efficiency of this response. Moreover, we observed that alternative inhibition of calpain via PKA, upon adenosine receptor activation, triggered a similar response of increased branching and consequently decreased speed of migration. This has direct implications in macrophage function, as adenosine acts as an anti-inflammatory signal and induces alternative polarization (i.e., M2 phenotype), which is associated with tissue repair and reduced macrophage motility (*37*).

In addition, we found that the variations in cell shape were larger and faster in macrophages than in endothelial cells. These findings might have implications regarding cell volume control, which also affects cell migration (*38*–*41*). Fast migrating macrophages would then require equally faster adaptation to the microenvironment and therefore need rapid changes in the function of molecules involved in cell volume control. Therefore, in macrophages and other fast migrating cells, we anticipate a prominent role for different cell volume regulators, such as mechanosensitive channels, transporters/exchangers, and pumps (*39*–*41*), which might contribute to provide the shape plasticity necessary for the efficient migration of these cells. Ion channels may also be essential for a mechanism that cells can use to release themselves from being trapped at junctions and to restart motility, which is to undergo a calcium-mediated reset of the actin cytoskeleton (*42*). This mechanism could play a pivotal role once macrophages have an increased branching and enter the slow-mode regime described by our model. Future studies should be performed to evaluate these hypotheses. In addition, microtubules and myosin II–dependent contractility contribute to the stability and lifetime of cellular protrusions (*25*, *43*). The incorporation of these components is feasible and will certainly expand the features of our model, but it is beyond the scope of the present work.

The model presented in this work provides us with the framework to explore the complex migration of bifurcated cells at the subcellular level and to reveal the details of cytoskeletal activity during this process. This model extends the current conceptual and predictive frameworks (*8*, *21*, *33*) to study cell migration inside 2D and 3D networks composed of linear tracks linked in different complex geometries. In addition, the present model can be used to study how these geometries affect the ability of cells to perform chemotaxis and haptotaxis in response to external gradients (*44*). This will be most relevant for cells migrating through dense tissues while guided by external signals, such as immune cells (*24*), cancer cells (*1*), and others.

## MATERIALS AND METHODS

### Experimental design

#### Cell culture

*Macrophages*. Bone marrow–derived macrophages were differentiated from myeloid precursors to macrophages in the span of 7 days. Briefly, bones were collected under protocol ORG 1062 granted to P.J.S. by the Animal Welfare Officers of the University Medical Center Hamburg-Eppendorf (UKE). Bone marrows were isolate as previously described (*45*), and the precursors were isolated and plated in Corning culture dishes of 100 mm in diameter using RPMI 1640, GlutaMAX supplement, and Hepes [10% fetal bovine serum (FBS)] (Gibco, Thermo Fisher Scientific), supplemented with 1% penicillin-streptomycin (PenStrep; Thermo Fisher Scientific) and macrophage colony-stimulating factor (100 μg/ml; Bio-Techne GmbH). The cells were incubated at 37°C and 5% CO_2_ for 7 days, adding 5 ml of RPMI 1640, GlutaMAX supplement, and Hepes (10% FBS), supplemented with 1% PenStrep, and macrophage colony-stimulating factor (100 μg/ml) on days 3 and 5. On day 7, cells were detached using 5 mM EDTA (Thermo Fisher Scientific) and subsequently plated in the micropatterns.

*Human umbilical vein endothelial cells*. The culture of HUVECs was performed as described previously (*8*). Briefly, HUVECs were acquired from PromoCell (C-12203) and used between passages 5 and 12. These cells were originally isolated from the vein of the umbilical cord of pooled donors. HUVECs were cultured in full endothelial growth medium that included the basal medium and the SupplementMix (PromoCell), supplemented with 1% PenStrep (Thermo Fisher Scientific) on 100-mm-diameter Corning culture dishes coated with fibronectin from bovine plasma (1 μl/ml) for 20 min at 37°C and 5% CO_2_. The dishes were washed once with Dulbecco’s phosphate-buffered saline (DPBS; 1×, Gibco, Thermo Fisher Scientific) before the culture. Once cultured, the cells were incubated at 37°C and 5% CO_2_ for 2 days until reaching a confluency of 80%. Once the monolayer is formed, the cells were passaged by detaching them with 3 ml of TrypLE Express (with Phenol Red, 1×, Gibco, Thermo Fisher Scientific) for 2 min and then plating 1.5 × 10^6^ cells/ml in new coated Corning culture dishes with 100 mm in diameter in a total of 10 ml of new full endothelial growth medium.

#### Micropatterning

The micropatterning was done using photopatterning using the Digital Micromirror Device (PRIMO, Alvéole) coupled to a T2i Eclipse microscope (Nikon). A polydimethylsiloxane stencil with a circle area of 5 mm was placed inside a bottom glass dish (FluoroDish, World Precision Instruments), which is a 35-mm-diameter glass bottom dish, previously cleaned with the Plasma Cleaner (PDC-32G-2, Harrick Plasma) for 5 min. Afterward, the area within the stencil was coated for 1 hour at room temperature with graft co-polymer of Poly(L-lysine)-g-poly(ethylene glycol) (PLL-g-PEG) (1 mg/ml), in the case of the HUVECs, or fluorescent PEG atto 633 (SuSoS AG), in the case of macrophages. For HUVECs and macrophages, the remaining pLL-PEG was washed with sterile water. Then, in both cases, 10 μl of photoactivator (PLPP, Alvéole) was added and then degraded using ultraviolet illumination at a power of 600 mJ/mm^2^, with the shape of the desired pattern. The pattern consisted of a hexagonal array, with a side of 60 μm and a width between hexagons of 20 μm in the case of HUVECs and a side of 12 μm and a width between hexagons of 4 μm in the case of macrophages. These patterns were created using the software Inkscape 1.0.2-2 (see section S6 and fig. S4). For HUVECs, the photoactivator (PLPP, Alvéole) was washed with 1× DPBS, and then the dish was coated for 20 min at 37°C with fibronectin (1 μl/ml) mixed with fibrinogen (1:10) to visualize the patterns and then rinsed with 1× DPBS. For macrophages, the remaining photoactivator (PLPP, Alvéole) was washed out with 1× DPBS and was left uncoated to let the cells adhere to the glass of the bottom of the dish. After this, 1× DPBS was removed and replaced with the corresponding medium. Last, the cells were plated as it follows. A total of 10,000 HUVECs were seeded and left overnight (around 16 hours) at 37°C and 5% CO_2_ to ensure their adhesion to the plate. In the case of macrophages, 10,000 cells were plated and imaged around 6 to 8 hours later.

#### Transfection

HUVECs were cultured until reaching a confluence of 80% and detached as mentioned in the “Cell culture” section. Then, the cells were transfected using the 4D-Nucleofector X Unit (Lonza) with 1 μg of F-tractin green fluorescent protein (GFP) plasmid (#58473, Addgene) and the P5 Primary Cell 4D-Nucleofector X Kit, electroporated corresponding to the transfection program (CA-167), and kept in culture overnight (around 16 hours) before use.

#### Live-cell imaging

The imaging for the phase contrast movies was performed using an inverted microscope (Leica Dmi8) equipped with an apochromat (APO) 10×/0.45 PH1, FL L 20×/0.40 CORR PH1, and APO 40×/0.95 objectives. Images were recorded with an ORCA-Flash4.0 Digital camera (Hamamatsu Photonics) using the MetaMorph version 7.10.3.279 software (Molecular Devices). The actin fluorescent videos were performed with a Nikon Eclipse TiE equipped with a 40× Plan Fluor Phase objective. Images were recorded with an Photometrics Prime 95B (back-illuminated scientific complementary metal-oxide semiconductor, 11-μm pixel size, 1200 pixels by 1200 pixels) connected to a Yokogawa CSU W-1 SoRa in dual-camera configuration connected to a confocal disk with 50-μm pinholes using the VisiView v4 software. During every acquisition, cells were kept at 37°C and 5% CO_2_. For HUVECs, the acquisition was done with the FL L 20×/0.40 CORR PH1; actin visualization was done with 40× Plan Fluor Phase objective of the Nikon Eclipse TiE, both imaged with a time interval of 3 min. The rest of the phase contrast movies were imaged with the APO 40×/0.95 objective with a time interval of 30 s. For macrophages, the acquisition was done with an APO 40×/0.95 objective, with a time interval of 30 s or 3 min. Treatment with 50 μM PD150606 calpain inhibitor was added just before acquisition, and 10 μM NECA (nonhydrolizable adenosine analog) was added 30 min before acquisition. 2D imaging was performed every 30 s or 30 min using a 20×/0.40 CORR PH1 objective.

#### Quantification of actin dynamics

Custom image analysis tools were developed to analyze actin distribution in relation to cell movement decisions at junction points. Segmentation was followed by manual correction for shape accuracy. Regions of interest corresponding to the cellular branches were defined and processed to create binary masks with the same dimensions as the original images. For each identified arm, the mask was rotated, such that the direction of cell movement always pointed rightward. For cells that traversed the same region multiple times in different directions, separate subregions were defined with appropriate orientation adjustments to ensure consistent analysis. Then, the area and actin content were quantified. For each frame, the total number of nonbackground pixels was quantified to determine the arm total area. The rightmost edge of the mask was identified as the leading edge in the direction of movement, and a “rightmost region” was defined as the 20 pixels extending inward from this edge. We measured actin activity in this region via a weighted actin sum calculated by sampling the original fluorescence image intensity at each pixel location, normalizing each value to a 0-1 scale (by dividing by 255), and summing these values.

In parallel, we manually labeled the behavioral role of each arm per frame. Branches were classified as “winning arm” (the one the cell choses to move into), “losing arm” (the one not chosen), and “back” (cell rear, where the cell came from). For macrophages, an additional classification was used: “exploring” (when cells remained at junctions without making a clear directional decision). This classification provided context for interpreting the actin distribution patterns observed. Actin measurements were normalized within each arm region to enable direct comparison, and a rolling window averaging approach (window size = 3) was applied to smooth temporal variations while preserving meaningful trends.

#### Tracking and neural networks

The coordinates of the tips of the cells across the junctions were monitored using neural networks with the ResNet50 architecture, as previously described (*46*, *47*). These networks were trained to analyze the movement of the cells and extract the coordinates of the tips. For labeling the movies, different frames were extracted from the movies in regular quantities of 12 units. This means that if the video was 120 frames long, then the 120 would be divided by 12, and the frames would be extracted 10 by 10 accordingly. Labels were added manually in both the tips of the cells, as well as the junctions, in each of the extracted frames. For HUVECs, a neural network was developed for all the movies, as the similarities between the movies allowed the generalization of the analysis, training a network on the labeled data for 175,000 iterations. For macrophages, neural networks were developed per single movie, and each neural network was trained during 300,000 iterations on the labeled data, which provided a reliable basis for each macrophage movie analyzed. The data provided by the neural networks consist of three columns per label (*X* and *Y* position and the likelihood of the prediction) and a row per frame. These data were represented in a video using a cutoff, supervising the predictions and correcting them manually when the prediction was not in the tip of the cell. In addition, the predictions for the position of the junctions, as they were a fixed point, were substituted by the exact *X* and *Y* position of each junction. To obtain the junction residency time, the following steps were followed. Frame by frame, the number of junctions in which the cell was spanning was annotated. With this information, the frequency of the amount of frames in which the cell was spanning a certain quantity of junctions was extracted. Last, these distributions of the junction residency times were compared with those obtained from the simulation data.

#### Cell length and C.O.M. speed measurements

Two complementary analyses were performed: cell length measurement and calculation of C.O.M. speed. The cell length analysis scripts processed files containing *X*-*Y* coordinate positions of cell tips and junction points. For each frame, the scripts identified distinct junctions (represented as central points A0, B0, etc.) and their associated cell tips (A1, A2, B1, B2, etc.). Euclidean distances were calculated between each junction point and its corresponding cell tips to determine the total cell length measurements. When cells spanned multiple junctions simultaneously, a penalty factor was applied to avoid double-counting overlapped distances, providing an accurate representation of the cell morphology. The C.O.M. velocity analysis calculated the displacement of the C.O.M. between consecutive frames. For each frame, a binary mask of the cell was created, and then the C.O.M. was obtained. Next, the displacement between consecutive frames was calculated, and, given the time interval for each movie, velocity of the C.O.M. was obtained.

#### Zebrafish in vivo experiments

The zebrafish (*Danio rerio*) for the imaging experiments were kept in a UK Home Office–approved facility. The standards of the Animal (Scientific Procedures) Act UK 1986 were followed for the experiments. The zebrafish larvae were between 3 and 5 days postfertilization for the experiments. The transgenic line Tg(mpx:EGFPi114; MPEG1:mCherry) was used to monitor the migratory behavior of macrophages (*48*). Briefly, adult fish were kept at 28.5°C in a 14:10-hour light:dark cycle. Eggs were collected and kept in 90-mm petri dish with methylthioninium chloride (methylene blue at 0.5 mg/ml). The fertilized eggs were also kept at 28.5°C until the end of the experiment. Imaging was performed using a six-well plate in which the larvae were kept anaesthetized with 4.2% (v/v) tricaine and embedded in agarose. The 1.5% agarose was prepared and allowed to cool before mounting the larvae to avoid any damage to the larvae. Once the larvae were mounted and the agarose was set, the well was filled with methylene blue and 4.2% (v/v) tricaine to avoid dehydration throughout. The plate was placed in the stage of the EVOS FL Auto2, and the humidifier function was used to set the temperature to 28.5°C. The ×10 magnification was used for the time-lapse function, where an image was taken every 3 to 5 min over the course of 20 to 24 hours. The images were acquired every 3 min using the bright-field filter as well as the GFP and the Texas red filters in the microscope

#### Code availability

Custom-built Fiji ImageJ macros, R scripts, and Python scripts are available from the corresponding authors upon request.
