# Supplementary material for: Trade-off between branching and polarity controls decision-making during cell migration
Source: Sci Adv. 2026 Jan 1;12(1):eads2734. doi: 10.1126/sciadv.ads2734 (PMC12757058; doi:10.1126/sciadv.ads2734)
Supplement: Supplementary file 1 — Supplementary Text Figs. S1 to S11 Table S1 Legends for movies S1 to S26 [file sciadv.ads2734_sm.pdf]

Science Advances
AAAS

Supplementary Materials for

Trade-off between branching and polarity controls decision-making during cell migration

Jiayi Liu *et al.*

Corresponding author: Nir S. Gov, nir.gov@weizmann.ac.il; Pablo J. Sáez, p.saez@uke.de

Sci. Adv. **12**, eads2734 (2026)
DOI: 10.1126/sciadv.ads2734

The PDF file includes:

Supplementary Text
Figs. S1 to S11
Table S1
Legends for movies S1 to S26

Other Supplementary Material for this manuscript includes the following:

Movies S1 to S26

Supplementary Text

S1. Steady-state actin treadmilling flows and polarity cue distribution profile

Calculation of the actin treadmilling flows

We assume that the local actin treadmilling flow emanating from the free edge of an arm splits symmetrically at each junction. For example, for a cell spanning 2 junctions, the flow from arm 1, v_1, splits symmetrically into arm 2 and the segment between node P and node Q (fig. S1A). Considering v_i of all the arms, the net actin treadmilling flow u_i within each arm is given by the sum of the flows that enter that arm

$$\begin{aligned} u_1 &= v_1 - \frac{v_2}{2} - \frac{v_3}{4} - \frac{v_4}{4} \\ u_2 &= v_2 - \frac{v_1}{2} - \frac{v_3}{4} - \frac{v_4}{4} \\ u_3 &= v_3 - \frac{v_4}{2} - \frac{v_1}{4} - \frac{v_2}{4} \\ u_4 &= v_4 - \frac{v_3}{2} - \frac{v_1}{4} - \frac{v_2}{4} \end{aligned} \quad \text{(S1)}$$

with the convention that a positive flow is away from the arm tip (fig. S1A). The middle segment that connects the two adjacent junctions P and Q has a net flow from all the incoming segments that connect to it

$$u_m = \frac{v_3}{2} + \frac{v_4}{2} - \frac{v_1}{2} - \frac{v_2}{2} \quad \text{(S2)}$$

and we take it to be positive towards junction P. Generally, the net actin flow of a free arm i is given by

$$u_i = v_i - \sum_{j \neq i} \frac{v_j}{2^{m_{i,j}}} \quad \text{(S3)}$$

where $m_{i,j}$ is the number of the junction nodes between arm i and arm j. Note that the relation $\sum_{j \neq i} \frac{v_j}{2^{m_{i,j}}} = 1$ always holds.

The net actin flow of a node connection segment k that connects two adjacent junction nodes Q and P is given by

$$u_k = \sum_{i\ connect\ to\ Q} \frac{v_i}{2} - \sum_{j\ connect\ to\ P} \frac{v_j}{2} \quad \text{(S4)}$$

where $\{i\}$ and $\{j\}$ denote the free arms that emanate from junction Q and P, respectively. The positive flow of u_k is taken to be along the direction from Q to P.

Note that within the cell, due to the nucleus and other organelles, the actin does not freely flow along the entire length of the cell, as suggested by our model (fig. S1A). However, these flows

drive the formation of an inhomogeneous concentration profile of the cytoplasmic contents (proteins as well as larger organelles) along the cell length, and this is captured by our simplified model.

Concentration profile of the polarity cue (actin polymerization inhibitor)

We consider a polarity cue *c(x)* diffusing in the cytoplasm, which is advected by the net actin treadmilling flows and acts as the inhibitor of actin polymerization. We assume that the concentration profile of the polarity cue relaxes faster than the timescale of the changes in the actin flow or cell shape [2-4]. This assumption greatly simplifies the problem and allows us to use the steady-state distribution for this concentration field, which is updated as the actin flows and cell shape changes. In the steady state, the advection-diffusion equation of the polarity cue within each arm is

$$\frac{\partial}{\partial x}\left(u_i\, c_i(x) + D\,\frac{\partial c_i(x)}{\partial x}\right) = 0 \qquad \text{(S5)}$$

where u_i is the net actin flow in arm i, $c_i(x)$ is the concentration of the polarity cue and D is the diffusion coefficient of the polarity cue. This gives a solution

$$c_i(x) = c_{i,0}\, \exp\left(-\frac{u_i x}{D}\right) + c_{i,1} \qquad \text{(S6)}$$

By applying a no-flux boundary condition at the ends of the arms, i.e.,$\left(u_i c_i(x) + D\,\frac{\partial c_i(x)}{\partial x}\,|_{x=x_i} = 0\right)$, we obtain the coefficient $c_{i,1} = 0$. The relationship between the coefficients $c_{i,0}$ (i=1,2,...,N) is calculated based on the continuity of the concentrations at the junction nodes, and it depends on the cell shape. For example, for a cell spanning 2 junctions, we denote node P as the reference point of polarity cue concentration with the concentration as c_0 (fig. S1B), and then the concentration profiles for all the arms and the middle segment can be written as

$$c_{1/2}(x_{1/2}) = c_0\, \exp\left(-\frac{u_{1/2}\, x_{1/2}}{D}\right)$$

$$c_m(x_m) = c_0\, \exp\left(-\frac{u_m\, x_m}{D}\right) \qquad \text{(S7)}$$

$$c_{3/4}(x_{3/4}) = c_0\, \exp\left(\frac{-u_m\, d}{D}\right) \exp\left(-\frac{u_{3/4}\, x_{3/4}}{D}\right)$$

The corresponding graphs are showed in fig. S1B.

The total concentration of the polarity cue is conserved within the cell

$$c_{tot} = \int_0^d c_m(x_m)\; dx_m + \sum_{j=1}^{4} \int_0^{x_j} c_j(x_j)\; dx_j \qquad \text{(S8)}$$

where the sum over $\{j\}$ represents the contributions of the free arms. By substituting Eq. S7 into Eq. S8, we obtain c_0 as

$$c_0 = \frac{c_{tot}}{D}\left[\frac{1-\exp\left(-\frac{u_m d}{D}\right)}{u_m} + \frac{1-\exp\left(-\frac{u_1 x_1}{D}\right)}{u_1} + \frac{1-\exp\left(-\frac{u_2 x_2}{D}\right)}{u_2} + \exp\left(-\frac{u_m d}{D}\right)\left(\frac{1-\exp\left(-\frac{u_3 x_3}{D}\right)}{u_3} + \frac{1-\exp\left(-\frac{u_4 x_4}{D}\right)}{u_4}\right)\right]^{-1} \quad \text{(S9)}$$

Generally, one can arbitrarily choose the reference point of the concentration field, denoted by c_0, at one of the junctions. Then the concentration in segment *i* (*i* could be either the free arm or the node connection segment) can be written as

$$c_i(x) = c_0 \exp\left(-\frac{\sum_k u_k d}{D}\right) \exp\left(-\frac{u_i x}{D}\right) \quad \text{(S10)}$$

where $\{k\}$ denotes the node connection segments between the segment *i* and the reference point. The direction of u_k is towards the reference point.

The total amount of the polarity cue is conserved within the cell

$$c_{tot} = \sum_l \int_0^d c_l(x_l)\ dx_l + \sum_j \int_0^{x_j} c_j(x_j)\ dx_j \quad \text{(S11)}$$

where $\{l\}$ represent the nodes connected by full segments (of length *d*) and $\{j\}$ represent the free arms. From Eqs. S10, S11 the value of c_0 can be found

$$c_0 = \frac{c_{tot}}{D}\left[\sum_l exp\left(-\frac{\sum_{k,l} u_{k,l} d}{D}\right)\frac{1-exp\left(-\frac{u_l d}{D}\right)}{u_l} + \sum_j exp\left(-\frac{\sum_{k,j} u_{k,j} d}{D}\right)\frac{1-exp\left(-\frac{u_j x_j}{D}\right)}{u_j}\right]^{-1} \quad \text{(S12)}$$

where the summation *k*, *l/j* is for the nodes connected by segments $\{k\}$ that are between the segment *l/j* and the reference point.

By substituting the expression of the inhibitor concentration (Eq. S10) into the expression of the steady-state local actin flows (Eq. 5), we obtain an implicit equation group of v_i (i = 1, 2, ..., N)

$$v_i = \beta \frac{1}{1 + c_0\, exp\left(-\frac{\sum_k u_k d}{D}\right) exp\left(-\frac{u_i x}{D}\right)} \quad \text{(S13)}$$

where u_i, u_k and c_0 is given by Eq. S3, Eq. S4 and Eq. S12, respectively. By numerically solving Eq. S13, we can determine the values of v_i corresponding to a specific set of $\{x_i\}$.
For the 2-junction case, the equation group of v_i (i = 1, 2, 3, 4) is obtained by substituting Eq. S7 into Eq. 5

$$v_{1/2} = \beta\left(1+\frac{c}{D}\frac{exp\left(-\frac{u_{1/2}x_{1/2}}{D}\right)}{\frac{1-exp\left(-\frac{u_m d}{D}\right)}{u_m}+\frac{1-exp\left(-\frac{u_1 x_1}{D}\right)}{u_1}+\frac{1-exp\left(-\frac{u_2 x_2}{D}\right)}{u_2}+exp\left(-\frac{u_m d}{D}\right)\left(\frac{1-exp\left(-\frac{u_3 x_3}{D}\right)}{u_3}+\frac{1-exp\left(-\frac{u_4 x_4}{D}\right)}{u_4}\right)}\right)^{-1}$$

$$v_{3/4} = \beta\left(1+\frac{c}{D}\frac{exp\left(-\frac{u_{3/4}x_{3/4}}{D}\right)exp\left(-\frac{u_m d}{D}\right)}{\frac{1-exp\left(-\frac{u_m d}{D}\right)}{u_m}+\frac{1-exp\left(-\frac{u_1 x_1}{D}\right)}{u_1}+\frac{1-exp\left(-\frac{u_2 x_2}{D}\right)}{u_2}+exp\left(-\frac{u_m d}{D}\right)\left(\frac{1-exp\left(-\frac{u_3 x_3}{D}\right)}{u_3}+\frac{1-exp\left(-\frac{u_4 x_4}{D}\right)}{u_4}\right)}\right)^{-1} \quad \text{(S14)}$$

where we have rescaled $c = \frac{c_{tot}}{c_s}$, and the actin flow speeds u_i are given by Eq. 7 and Eq. 8

S2. Critical local actin polymerization activity for migration

Critical local actin polymerization activity for symmetry breaking

As in our previous model for cells on one junction, for cells that are spanning symmetrically on more junctions, there are also critical values of β which decide whether the cell can break the symmetry and start migrating. The β_c is determined by two lengths of the cell: the critical length of polarization due to the force balance, L_p, and the critical length of polarization due to the redistribution of the polarity cue, L_c.

L_p is calculated by the balance between the protrusion force and the elasticity of the cell. The cell has a tendency to elongate when its length is shorter than L_p. For the cases where cells occupy multiple nodes, the expression of L_p is the same as when cells migrate on a one-dimensional line, and when cells migrate on a single junction

$$L_p = \frac{1}{2}(1-c)+\frac{\beta}{2k}+\sqrt{c+\left(\frac{1-c}{2}+\frac{\beta}{2k}\right)^2} \quad \text{(S15)}$$

On the other hand, when the cell length is longer than L_c, the actin treadmilling flows of arms will deviate from the uniform solutions. In the instance of this symmetry breaking event, all the arms have the equal length, but there will be a small bias in some of the arms. We can derive L_c based on this event, and then the β_c is obtained by equating L_p and L_c.

For cells migrating on a one-dimensional line, and for cells on a single junction, the β_c is given by

$$\beta_c(n_j = 0) = \frac{D}{2c}+ck+\frac{ck^2}{2D}+\frac{D-ck}{2cD}\sqrt{D^2+2c(1+2c)Dk+c^2k^2} \quad \text{(S16)}$$

$$\beta_c(n_j = 1) = \frac{D}{c} + ck + \frac{ck^2}{4D} + \frac{2D-ck}{4cD}\sqrt{4D^2 + 4c(1+2c)Dk + c^2k^2} \quad \text{(S17)}$$

Next, we will derive the β_c in the 2-junction case as an example. We assume that $x_i = l$, $u_{1/2} = \epsilon$ and $u_{3/4} = -\epsilon$ (fig. S1A), then according to Eq. S1 and Eq. S2, we have $u_m = u_3 + u_4 = -2\epsilon$. By substituting these values to Eq. S14, we obtain the actin treadmilling flows equations

$$v_{1/2} = \beta\left(1 + \frac{c}{D}\frac{exp\left(-\frac{\epsilon l}{D}\right)}{\frac{1}{2\epsilon}\left[1-exp\left(-\frac{2\epsilon d}{D}\right)\right]+\frac{2}{\epsilon}\left[1-exp\left(-\frac{\epsilon l}{D}\right)\right]+exp\left(\frac{2\epsilon d}{D}\right)\frac{2}{\epsilon}\left[1-exp\left(\frac{\epsilon l}{D}\right)\right]}\right)^{-1}$$

$$v_{3/4} = \beta\left(1 + \frac{c}{D}\frac{exp\left(\frac{2\epsilon d}{D}\right)exp\left(\frac{\epsilon l}{D}\right)}{\frac{1}{2\epsilon}\left[1-exp\left(-\frac{2\epsilon d}{D}\right)\right]+\frac{2}{\epsilon}\left[1-exp\left(-\frac{\epsilon l}{D}\right)\right]+exp\left(\frac{2\epsilon d}{D}\right)\frac{2}{\epsilon}\left[1-exp\left(\frac{\epsilon l}{D}\right)\right]}\right)^{-1} \quad \text{(S18)}$$

Then we can write the global flow of arm 1 (Eq. S1) as

$$\epsilon = \frac{\beta}{2}\left(1 + \frac{c}{D}\frac{exp\left(-\frac{\epsilon l}{D}\right)}{\frac{1}{2\epsilon}\left[1-exp\left(-\frac{2\epsilon d}{D}\right)\right]+\frac{2}{\epsilon}\left[1-exp\left(-\frac{\epsilon l}{D}\right)\right]+exp\left(\frac{2\epsilon d}{D}\right)\frac{2}{\epsilon}\left[1-exp\left(\frac{\epsilon l}{D}\right)\right]}\right)^{-1}$$

$$-\frac{\beta}{2}\left(1 + \frac{c}{D}\frac{exp\left(\frac{2\epsilon d}{D}\right)exp\left(\frac{\epsilon l}{D}\right)}{\frac{1}{2\epsilon}\left[1-exp\left(-\frac{2\epsilon d}{D}\right)\right]+\frac{2}{\epsilon}\left[1-exp\left(-\frac{\epsilon l}{D}\right)\right]+exp\left(\frac{2\epsilon d}{D}\right)\frac{2}{\epsilon}\left[1-exp\left(\frac{\epsilon l}{D}\right)\right]}\right)^{-1} \quad \text{(S19)}$$

Expand it in the first order in ϵ

$$\epsilon = \frac{\beta c}{D}\frac{(d+l)(d+4l)}{(c+d+4l)^2}\epsilon + O(\epsilon^2) \quad \text{(S20)}$$

By solving it for l, we obtain the solution of the critical length for each arm

$$l_c = \frac{8dD + c\left(8D - 5d\beta + \sqrt{\beta(16cD - 48dD + 9d^2\beta)}\right\}}{8(\beta c - 4D)} \quad \text{(S21)}$$

The critical length of the cell is

$$L_c = 4l_c + d = \frac{8dD + c\left(8D - 5d\beta + \sqrt{\beta(16cD - 48dD + 9d^2\beta)}\right\}}{2(\beta c - 4D)} + d \quad \text{(S22)}$$

By equating L_p (Eq. S15) and L_c (Eq. S22), we obtain the β_c for symmetry breaking of cells occupying 2 junctions,

$$\beta_c(n_j = 2) = \frac{1}{2c(4D+3dk)}\Big[16D^2 + k^2c(c-3d)(3d+1) + 4Dk(2c^2 + 3d - 3cd) + (4D - kc - 3kd)\sqrt{16D^2 + 8c(2c-3d+1)Dk + c^2(3d+1)^2k^2}\Big\} \quad \text{(S23)}$$

By similar derivations, we obtain the β_c for the 3-junction case and for the 4-junction case (fig. S1C,D),

$$\beta_c(n_j = 3) = \frac{1}{24c(5D+6dk)} [400D^2 + 9k^2c(c-8d)(8d+1) + 120Dk(c^2+4d-4cd) + (20D-3kc-24kd)\sqrt{400D^2+120c(2c-8d+1)Dk+9c^2(8d+1)^2k^2}] \tag{S24}$$

$$\beta_{c4}^{(i)}(n_j = 4) = \frac{1}{8c(8D+9dk)} [64D^2 + k^2c(c-9d)(9d+1) + 8Dk(2c^2+9d-9cd) + (8D-kc-9kd)\sqrt{64D^2+16c(2c-9d+1)Dk+c^2(9d+1)^2k^2}] \tag{S25}$$

$$\beta_{c4}^{(ii)}(n_j = 4) = \frac{1}{86(4D+7dk)} [144D^2 + k^2c(c-21d)(21d+1) + 12Dk(2c^2+21d-21cd) + (12D-kc-21kd)\sqrt{144D^2+24c(2c-21d+1)Dk+c^2(21d+1)^2k^2}] \tag{S26}$$

Critical local actin polymerization activity for occupying multiple junctions

To obtain the critical β for cells to migrate when occupying multiple junctions, it is also necessary to consider if β is large enough to enable cells to achieve the minimum length to occupy a certain number of junctions. For a cell with N ($N \geq 3$) arms, i.e., spanning across $N-2$ junctions, the minimum length is

$$L_{min} = (N-3)d \tag{S27}$$

which is the total length of the segments between the adjacent junctions that the cell is occupying.

By equating the polarization length, L_p (Eq. S15), and L_{min} (Eq. S27), we obtain the critical β for cells to occupy $N-2$ junctions

$$\beta_d(n_j = N-2) = \frac{k[(N-3)d-1][(N-3)d+c]}{(N-3)d} \tag{S28}$$

In fig. S1F we plot β_c and β_d for different number of junctions, as well as the critical beta of the slow process in the one-junction case, for the range of grid size d that we used in this study. Note that the larger one between β_c and β_d is the critical β for cells to migrate when occupying multiple junctions. For the 2-junction case, the critical β is β_c, while for the 3-junction and 4-junction cases, the critical β is β_d, within the range of d values shown.

S3. Micropatterns of HUVEC and macrophages

The micropatterns of HUVEC and macrophages in our experiments has been performed as described the "Experimental Methods" section of the main text, and shown schematically in Fig. S2. Note that we have adapted the size of the hexagonal array to allow the HUVEC and macrophages to span simultaneously several junctions. Because macrophages are smaller than HUVEC we adapted the size of the hexagons previously used [8], as described in fig. S2.

S4. Mean-squared-displacement

The mean-squared-displacement (MSD) of the center-of-mass of cells is given by

$$MSD(t) = \langle x(t)^2 + y(t)^2 \rangle = \frac{1}{N_c}\sum_{i=1}^{N_c}(x_i(t)^2 + y_i(t)^2) \quad \text{(S29)}$$

where N_c is the number of cells that considered as an ensemble to be averaged (i.e., we run N_c repetitions of simulations), and $x/y(t)$ is the x/y coordinate of the centroid of ith cell (i = 1, 2, ..., N_c) at time t in the simulation. Here we set N_c = 1000.

S5. Junction residency time for a specific number of junctions

We show the $\beta - \sigma$ phase diagrams of the average junction residency time, $\langle T_{junc} \rangle$, for d = 3.0 and d = 3.5 in the main text. The corresponding phase diagrams of the mean junction residency time for a specific number of junctions, $\langle T_{junc}^{n_j} \rangle$, are shown in fig. S4A,B, respectively

S6. Hexagonal residency time

The hexagonal residency time, T_{hex}, is defined as the time from when the C.O.M of a cell enter a hexagon of the network, until it leaves it entering a new one. We show the phase diagram of the mean hexagonal residency time, $\langle T_{hex} \rangle$ (fig. S6A). Consistent with the trend of $\langle T_{junc} \rangle$ (Fig. 5), the increase of β decreases $\langle T_{hex} \rangle$.

We found that for cells with large β and small σ, the distribution of $\langle T_{hex} \rangle$ always have some peaks, which are correlated to some periodic patterns of cell length and number of junctions during the migration process. For example, the $\langle T_{hex} \rangle$ distribution for (β, d, σ)=(10.0,2.5,0.5) has two discrete peaks (blue/red in fig. S6B)), corresponding to the first and second half of the cycle of the number of junctions and cell length (blue and red lines in fig. S6B). We show the snapshots of the shape and the C.O.M. of the cell in a cycle. The C.O.M. is marked by the red dot, and the grid that it is occupying is colored in blue/red, which corresponds to the blue/red peak in fig. S6B. We can ignore the small peak near $T_{hex} \approx 0$ (gray peak in fig. S6B), which is not related to periodic patterns. They originate from the small fluctuation of arm lengths when the cell shape is nearly symmetric and the C.O.M. is close to the border of two adjacent grid. In this case, the slight drift of the C.O.M. may lead to the residency on either of two adjacent grids.

The peaks of T_{hex} distributions become less significant as β decreases (fig. S6C), because there are almost no oscillations in the cell length. The peaks in fig. S6B shift left and mix with each other when σ is large (fig. S6C), because the noise makes the C.O.M. drifts more irregularly.

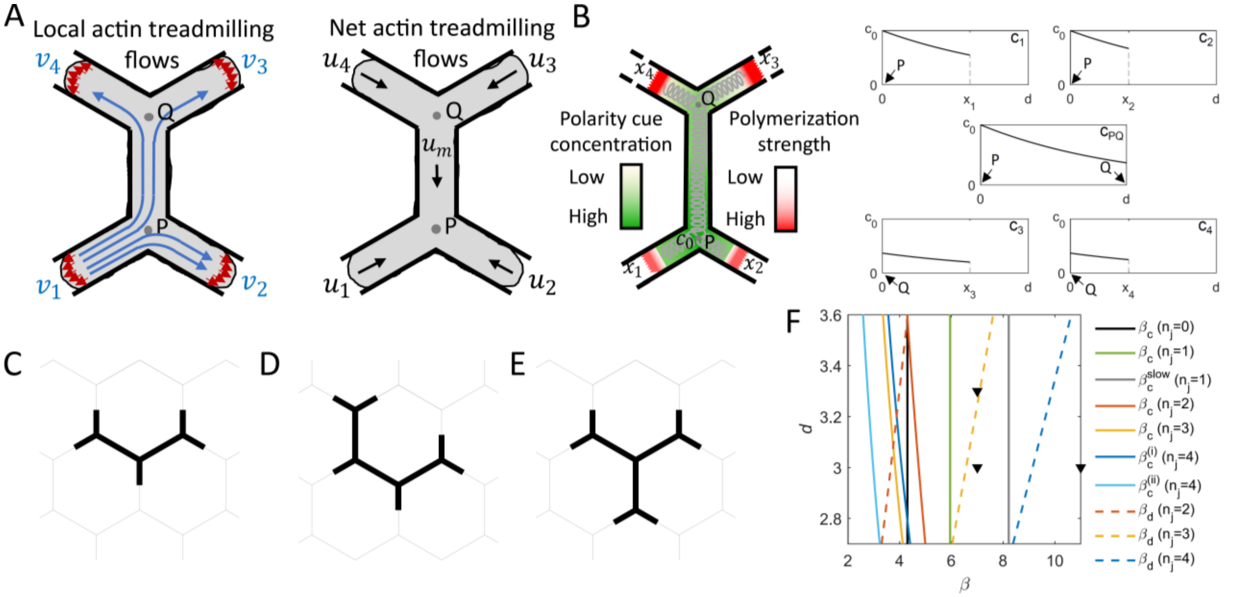

Fig. S1. Theoretical mechanism of branched cell polarization. (A) Schemes showing the local actin treadmilling flows at each edge of the arms, the split of the local actin flow of arm 1, and the net actin treadmilling flows in each segment of the cell. **(B)** Example of the concentration field of the polarity cue, which is affected by the advection flows. The cell elasticity is denoted by the grey springs. Polarity cue concentration profiles of all the segments *x*1-4 shown in the scheme, where *d* is the length of the hexagonal network segments. Node P is the reference point where the polarity cue concentration is fixed to be c_0 (Eq. S12), and node Q is the other junction node that the cell is spanning. **(C)** Cell shape while spanning 3 junctions. **(D,E)** Two possible cell shapes for cells spanning 4 junctions. **(F)** Critical β_s for symmetry breaking and for occupying multiple junctions (Eqs. S16, S17, S23, S24, S25, S26, S28). Black inverted triangles correspond to those in Fig. 2B. Shaded region indicates the range of (β, d) under which the cell becomes trapped across two junctions. Other key parameter: k = 0.8.

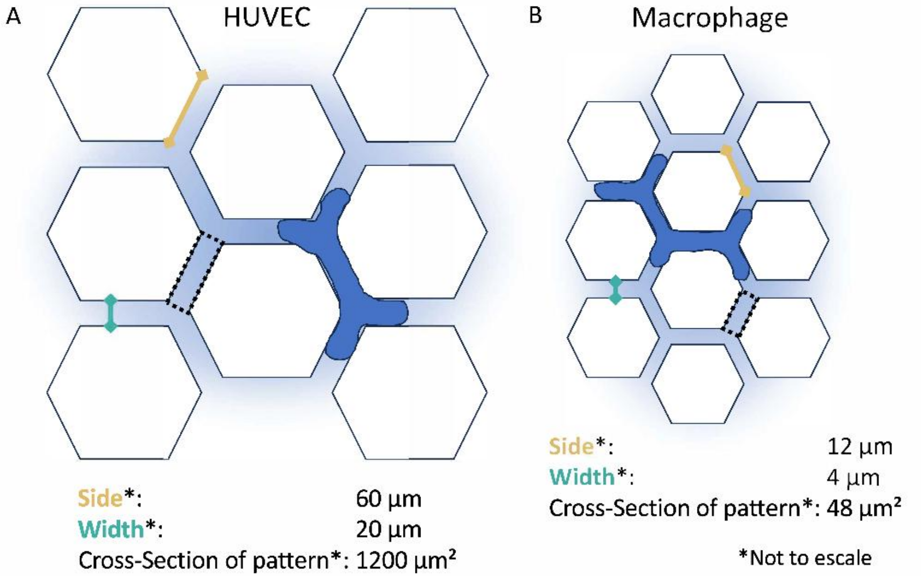

Fig. S2. Experimental design of micropatterns. **(A)** Hexagonal micropattern of the HUVEC. **(B)** Hexagonal micropattern of the macrophages. Note the differences in the size.

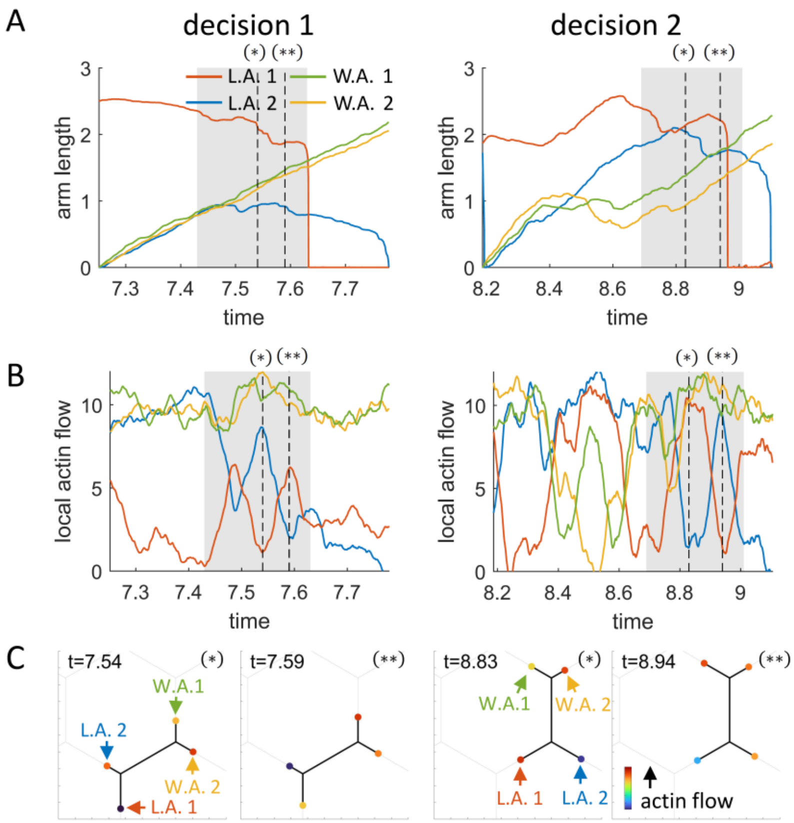

Fig. S3. Simulation of seesaw oscillations during directional decision-making by a highly branched migrating cell with macrophage-like parameters (movie S6). (A) Dynamics of arm length during two decision-making events. **(B)** Dynamics of local actin flow at the arm tips. **(C)** Snapshots of cell shape at selected time points marked by black dashed lines in A) and B). Branches are label to depict the winning and losing arms, W.A. and L.A., respectively. Key parameters: β = 10.0, d = 3.8, σ = 3.0, k = 0.8, r = 5.0.

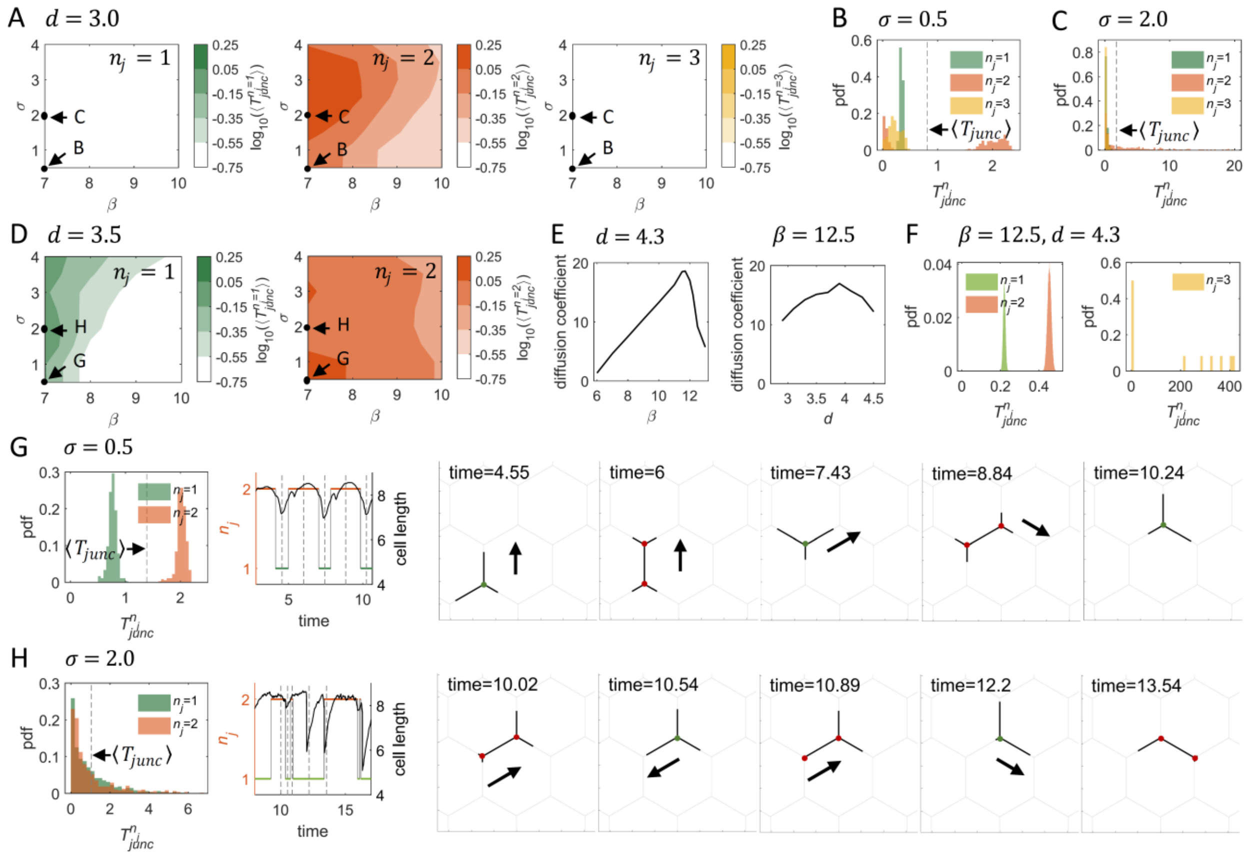

Fig. S4. Analysis of the junction residency time as function of noise and hexagonal network edge size. (A) $\beta{-}\sigma$ phase diagrams of the junction residency time for a specific number of junctions, $\langle T_{junc}^{n_j} \rangle$ (log scale) for d=3.0. **(C)** Distribution (probability density function, P.D.F.) of $T_{junc}^{n_j}$ for β = 7, d = 3.0, σ = 0.5, and **(C)** σ = 2.0. **(D)** β-σ phase diagrams of $\langle T_{junc}^{n_j} \rangle$ (log scale) for d=3.5 n_j = 1, and n_j = 2. **(E)** Diffusion coefficient as a function of β with d=4.3, and as function of d with β = 12.5. **(F)** Distribution (P.D.F.) of $T_{junc}^{n_j}$ for β =12.5, d = 4.3 for n_j = 1, n_j = 2, and n_j = 3. Maximal simulation time: T=50000. Other key parameters: k = 0.8, r = 5.0. **(G)** Distribution (P.D.F) of $\langle T_{junc}^{n_j} \rangle$ for β = 7, d = 3.5, and σ = 0.5. Dynamics of the number of junctions and cell length for β = 7, d = 3.5, σ = 0.5 / σ = 2.0 and corresponding snapshots corresponding to the time stamps (dashed lines). **(H)** Similar plots to those shown in G but for σ = 2.0

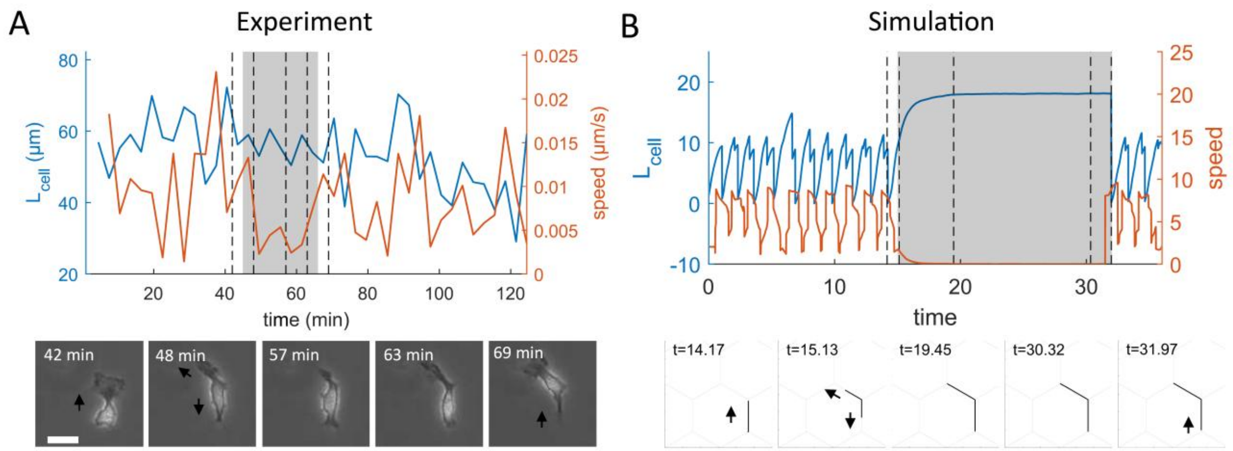

Fig. S5. Comparisons between slow-mode dynamics on large grids in simulations and experiments for a macrophage. (A) Dynamics of cell length and C.O.M. speed in an experiment of a macrophage moving in a hexagonal network. Snapshots at time points marked by dashed lines. Scale bar: 20 µm. **(B)** Corresponding simulation of macrophage, exhibiting a slow-mode event. Note that here one of the long arms grows back along the direction from which the cell arrived at the junction, which is different from the slow-mode behavior shown in Fig. 5H. Snapshots at time points marked by dashed lines. Key simulation parameters: (β, d, σ, k, r) = (13.0, 9.0, 1.0, 0.8, 5.0).

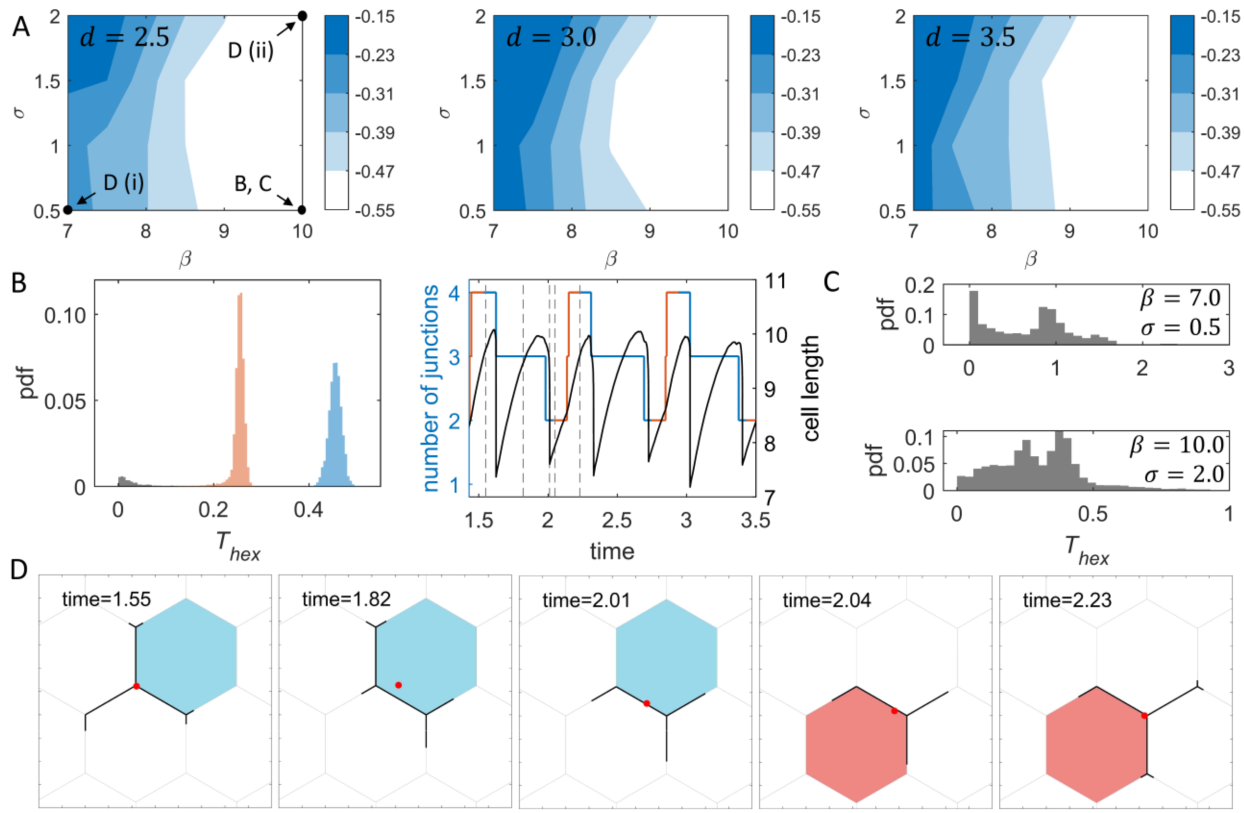

Fig. S6. Analysis of the simulated residency time within the hexagonal elements of the network. (A) $\beta - \sigma$ phase diagram of the hexagonal residency time, $\langle T_{hex} \rangle$, (log scale) for different grids sizes and correspond to the grid size d = 2.5, d = 3.0 and d = 3.5. **(B)** $\langle T_{hex} \rangle$ distribution in a simulation for (β, d, σ) = (10.0, 2.5, 0.5). Dynamics of the number of arms and cell length. Blue/red lines of the number of arms correspond to the blue/red peaks. **(C)** $\langle T_{hex} \rangle$ distribution for d = 2.5 and (β, σ) = (7.0, 0.5), and (β, σ) = (10.0, 2.0), respectively. Other key parameters: k = 0.8, r = 5.0. **(D)** Snapshots of cell shape in the simulation in B) corresponding to the time stamps marked by gray dashed lines. The red point denotes the centroid of the cell; colored hexagons shows where the centroid is located, and blue/red correspond to the blue/red peaks of $\langle T_{hex} \rangle$ in B).

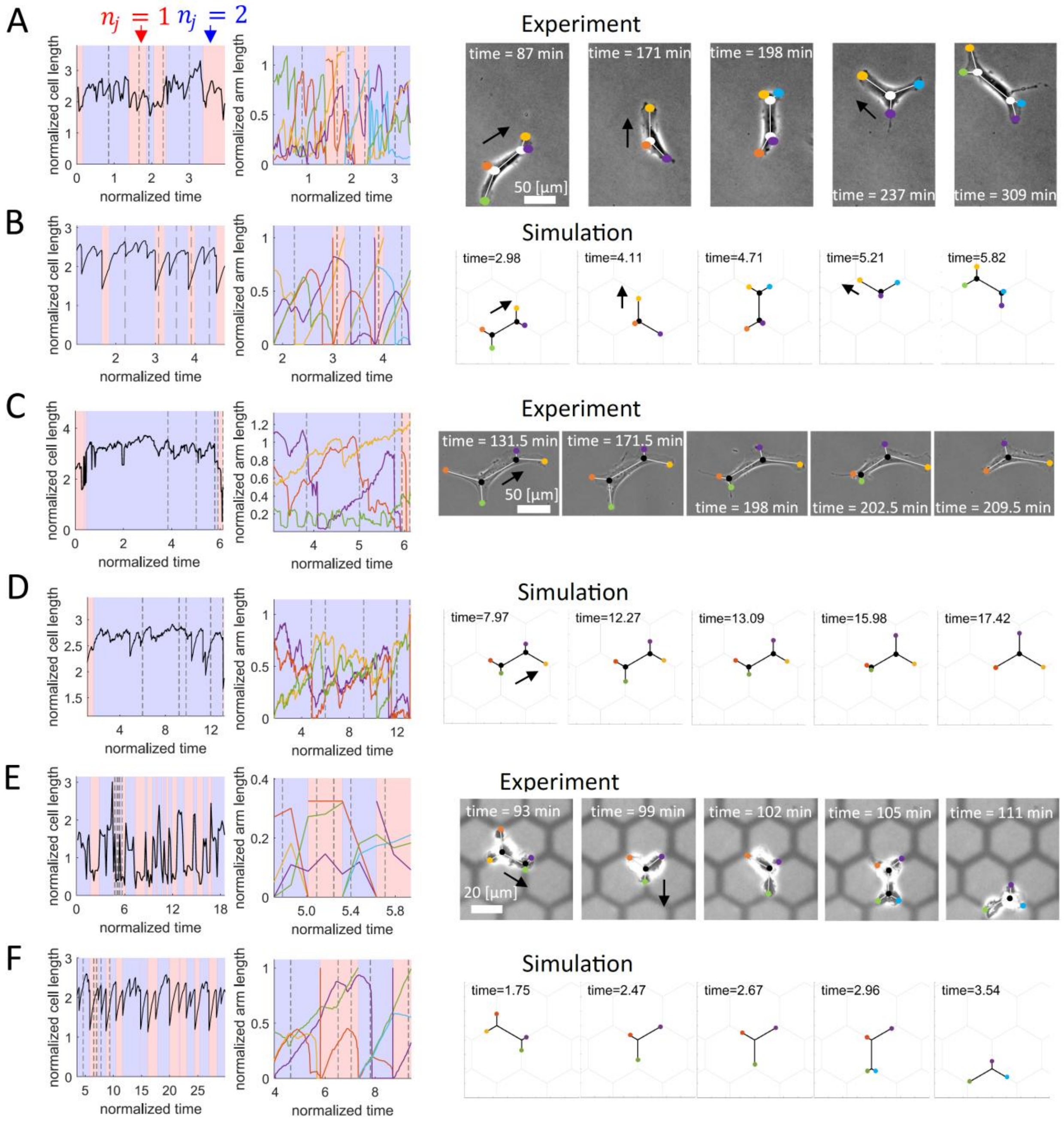

Fig. S7. Comparisons between cell shapes and migration patterns in simulations and experiments of HUVEC and macrophages. (A,C) Experiments on migrating HUVEC (A, movie S13; C, movie S15). **(B,D)** Simulations of non-migrating HUVEC (B, movie S14; D, movie S16). Dynamics of the normalized cell length and arm lengths and snapshots of the cell, corresponding to the gray dashed lines are shown. Key simulation parameters: (β, d, σ, k) = (9.0, 3.6, 1.8, 0.8) in (B), and (β, d, σ, k) = (7.0, 3.5, 2.7, 0.7) in (D). Other key parameter: r = 5.0. **(E)** Experiment on a migrating macrophage (movie S17). **(F)** Simulations of the migrating macrophage (movie S18). The dynamics of the normalized cell length and the arm lengths, and snapshots of the cell, corresponding to the gray dashed lines. Key simulation parameters: (β, d, σ) = (10.0, 4.0, 2.5). Other key parameters: k = 0.8, r = 5.0.

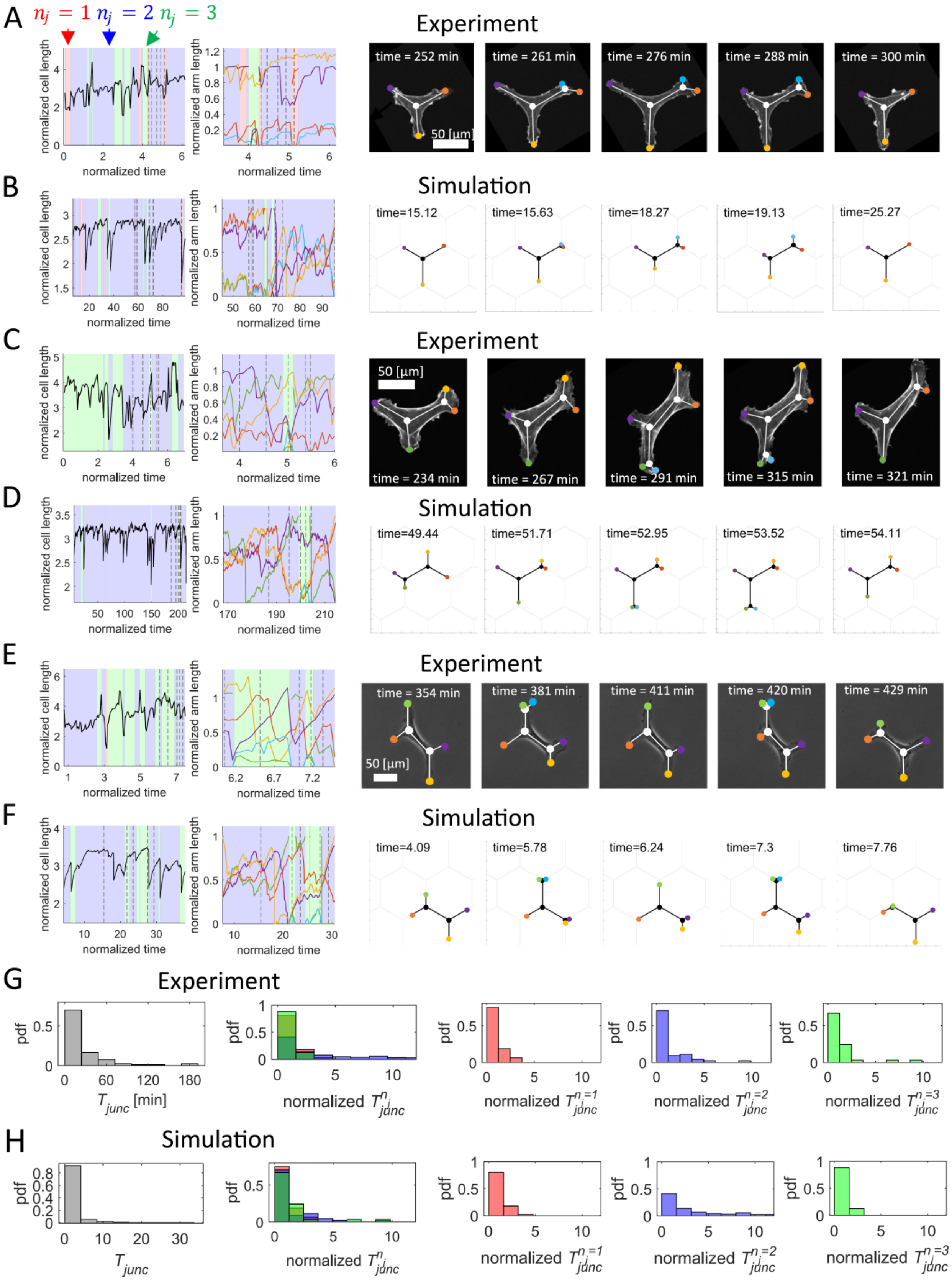
A
$n_j = 1$ $n_j = 2$ $n_j = 3$
Experiment
time = 252 min
time = 261 min
time = 276 min
time = 288 min
time = 300 min
50 [μm]
B
Simulation
time=15.12
time=15.63
time=18.27
time=19.13
time=25.27
C
Experiment
50 [μm]
time = 234 min
time = 267 min
time = 291 min
time = 315 min
time = 321 min
D
Simulation
time=49.44
time=51.71
time=52.95
time=53.52
time=54.11
E
Experiment
time = 354 min
time = 381 min
time = 411 min
time = 420 min
time = 429 min
50 [μm]
F
Simulation
time=4.09
time=5.78
time=6.24
time=7.3
time=7.76
normalized cell length
normalized arm length
normalized time
G
Experiment
H
Simulation
pdf
T_{junc} [min]
T_{junc}
normalized $T^{n_j}_{junc}$
normalized $T^{n_j=1}_{junc}$
normalized $T^{n_j=2}_{junc}$
normalized $T^{n_j=3}_{junc}$

Fig. S8. Comparisons between cell shapes dynamics in simulations and experiments for weakly motile HUVEC. (A,C,E) Experiments on non-migrating HUVEC (A, movie S19; C, movie S21; E, movie S23). **(B,D,F)** Simulations of non-migrating HUVEC (B, movie S20; D, movie S22; F, movie S24). Dynamics of the normalized cell length and arm lengths, and snapshots of the cell, corresponding to gray dashed lines are shown. Key simulation parameters: (β, d, σ) = (7.0, 3.0, 1.8) in (B), (β, d, σ) = (7.0, 2.7, 2.1) in (D), and (β, d, σ) = (7.6, 2.7, 2.0) in (F). Other key parameters: k = 0.8, r = 5.0. **(G)** Distribution (P.D.F.) of T_{junc} and distribution of normalized $T_{junc}^{n_j}$ in the experimental regime in which the cells spans 1, 2 or 3 junctions. A total of 3 cells across 1725 minutes were used to obtain these distributions. **(H)** Distribution of T_{junc} and distribution of normalized $T_{junc}^{n_j}$ from a long simulation with parameters of B). Simulation time: T = 1000.

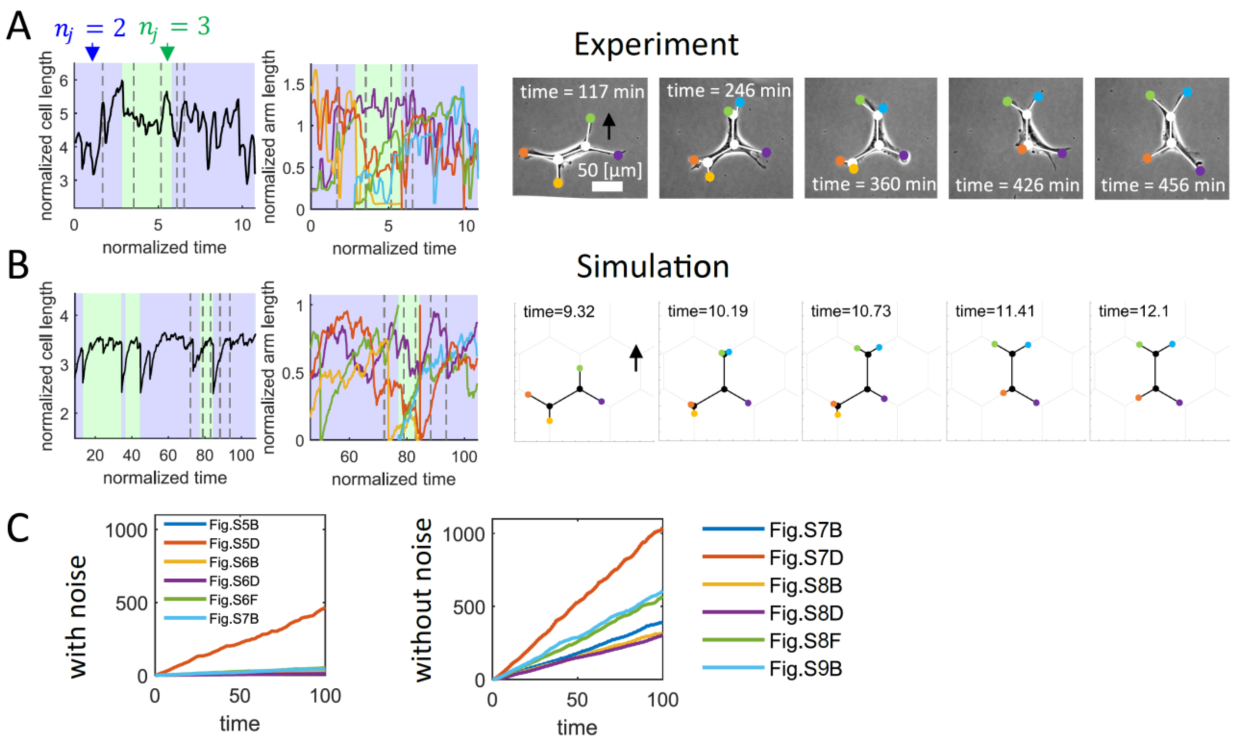

Fig. S9. Comparisons between cell shapes dynamics in simulations and experiments for HUVEC with larger cell-substrate adhesiveness. (A) Experiment on HUVEC treated by the inhibitor of calpain (movie S25). **(B)** Simulation of HUVEC with larger cell-substrate adhesiveness (movie S26). Dynamics of the normalized cell length and the arm lengths. Snapshots of the cell, corresponding to gray dashed lines. Key simulation parameters: (β, d, σ, r) = (8.5, 2.7, 2.5, 7.0). Other key parameters: k = 0.8. **(C)** Comparisons of *MSD* of the simulated HUVEC of fig. S7-S9, with the noise levels identical to those used in the corresponding simulations or without the noise.

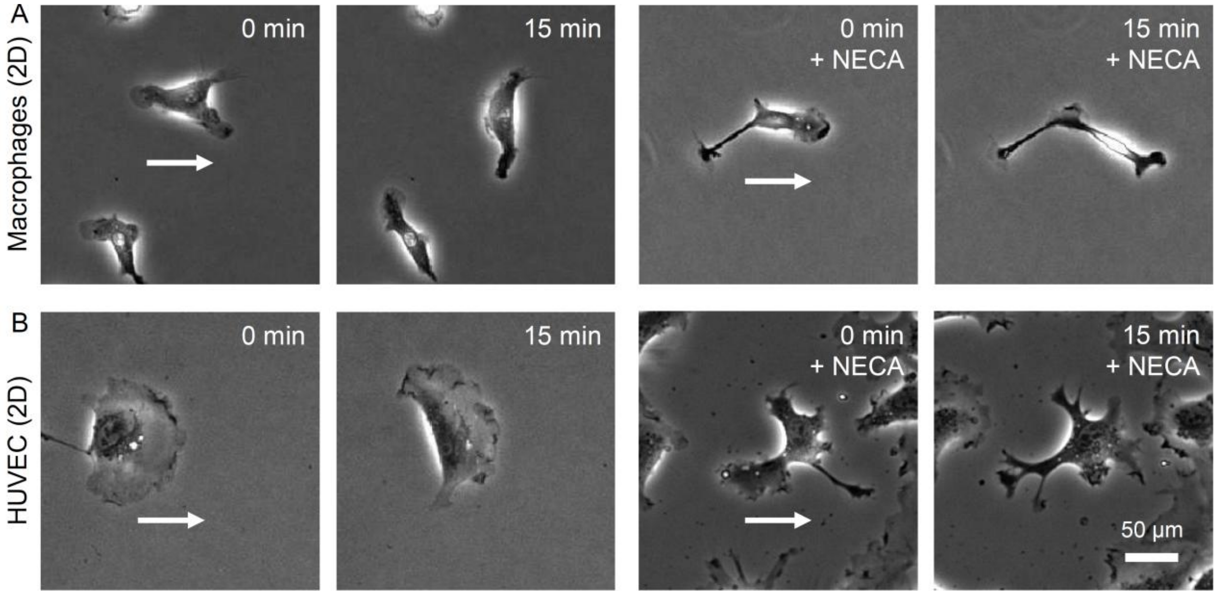

Fig. S10. Adenosine receptor activation increase adhesion and branching in 2D. (A) macrophages moving in a 2D free surface under control conditions or after treatment with 10 µM NECA at indicated time points. **(B)** same experiment in HUVEC. Scale bar is 50 µm.

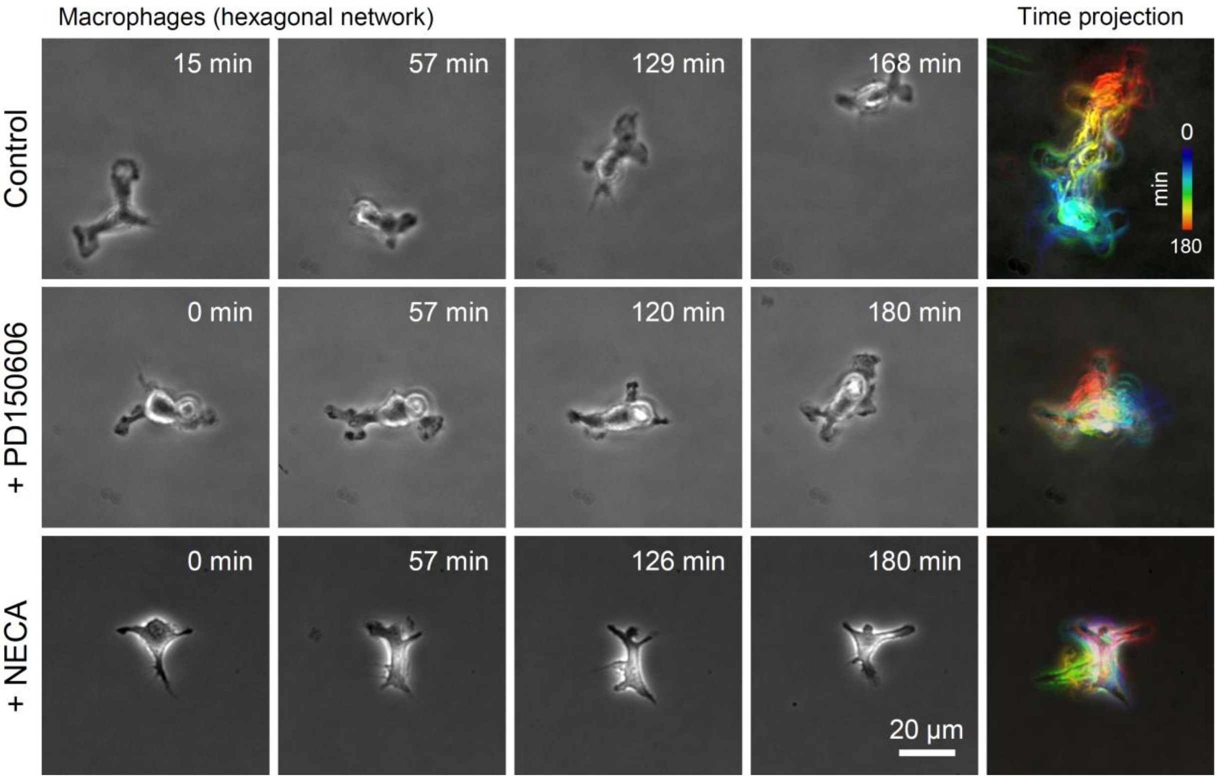

Fig. S11. Enhanced cell-substrate adhesion reduces long range exploration in macrophages. Images of macrophages moving on a hexagonal network at indicated time points and the corresponding color coded time projection (180 min). Macrophages were imaged under control conditions (top panel), 50 µM PD150606 (middle panel) and 10 µM NECA (bottom panel). Scale bar: 20 µm. Note the reduced motility under the treatments in comparison to the control.

Parameter	Value
c	3.85
D	3.85
f_s	5
κ	20
δ	250

Table S1. Parameters used in the model. List of parameters and values used in the model.

Additional supplementary files (movies S1-S26)

Movie legends

Movie S1: Macrophages migrating in vivo in the basal layer of keratinocytes in the zebrafish tailfin (Fig. 1A).
Movie S2: Macrophage migrating *in vivo,* and its skeletonized shape (Fig. 1B, and C).
Movie S3: Simulation of the migration of the same cell on grids with different sizes (Fig. 2B).
Movie S4: Simulation of a migrating HUVEC (Fig. 3A).
Movie S5: Experiment of a migrating HUVEC (Fig. 3B).
Movie S6: Simulation of a migrating macrophage (Fig. 4A).
Movie S7: Experiment of a migrating macrophage (Fig. 4B and Fig. 6G).
Movie S8: Experiment of a migrating HUVEC (Fig. 6A).
Movie S9: Simulation of the migrating HUVEC in Fig. 6A (Fig. 6C).
Movie S10: Simulation of the migrating macrophage in Fig. 6G (Fig. 6I).
Movie S11: Experiment of a migrating HUVEC treated by the inhibitor of calpain (Fig. 7A).
Movie S12: Simulation of the migrating HUVEC in Fig. 7A (Fig. 7C).
Movie S13: Experiment of a migrating HUVEC (fig. S7A).
Movie S14: Simulation of the migrating HUVEC in fig. S7A (fig. S7B).
Movie S15: Experiment of a migrating HUVEC (fig. S7C).
Movie S16: Simulation of the migrating HUVEC in fig. S7C (fig. S7D).
Movie S17: Experiment of a migrating macrophage (fig. S7E).
Movie S18: Simulation of the migrating macrophage in fig. S7E (fig. S7F).
Movie S19: Experiment of a weakly motile HUVEC (fig. S8A).
Movie S20: Simulation of the weakly motile HUVEC in fig. S8A (fig. S8B).
Movie S21: Experiment of a weakly motile HUVEC (fig. S8C).
Movie S22: Simulation of the weakly motile HUVEC in fig. S8C (fig. S8D).
Movie S23: Experiment of a weakly motile HUVEC (fig. S8E).
Movie S24: Simulation of the weakly motile HUVEC in fig. S8E (fig. S8F).
Movie S25: Experiment of a migrating HUVEC treated with the inhibitor of calpain (fig. S9A).
Movie S26: Simulation of the migrating HUVEC in fig. S9A (fig. S9B).
